# Transcriptomic responses of *Biomphalaria pfeifferi* to *Schistosoma mansoni*: Investigation of a neglected African snail that supports more *S*. *mansoni* transmission than any other snail species

**DOI:** 10.1371/journal.pntd.0005984

**Published:** 2017-10-18

**Authors:** Sarah K. Buddenborg, Lijing Bu, Si-Ming Zhang, Faye D. Schilkey, Gerald M. Mkoji, Eric S. Loker

**Affiliations:** 1 Department of Biology, Center for Evolutionary and Theoretical Immunology, University of New Mexico, Albuquerque, New Mexico, United States of America; 2 National Center for Genome Resources, Santa Fe, New Mexico, United States of America; 3 Center for Biotechnology Research and Development, Kenya Medical Research Institute, Nairobi, KEN; George Washington University School of Medicine and Health Sciences, UNITED STATES

## Abstract

**Background:**

*Biomphalaria pfeifferi* is highly compatible with the widespread human-infecting blood fluke *Schistosoma mansoni* and transmits more cases of this parasite to people than any other snail species. For these reasons, *B*. *pfeifferi* is the world’s most important vector snail for *S*. *mansoni*, yet we know relatively little at the molecular level regarding the interactions between *B*. *pfeifferi and S*. *mansoni* from early-stage sporocyst transformation to the development of cercariae.

**Methodology/Principal findings:**

We sought to capture a portrait of the response of *B*. *pfeifferi* to *S*. *mansoni* as it occurs in nature by undertaking Illumina dual RNA-Seq on uninfected control *B*. *pfeifferi* and three intramolluscan developmental stages (1- and 3-days post infection and patent, cercariae-producing infections) using field-derived west Kenyan specimens. A high-quality, well-annotated *de novo B*. *pfeifferi* transcriptome was assembled from over a half billion non-*S*. *mansoni* paired-end reads. Reads associated with potential symbionts were noted. Some infected snails yielded fewer normalized *S*. *mansoni* reads and showed different patterns of transcriptional response than others, an indication that the ability of field-derived snails to support and respond to infection is variable. Alterations in transcripts associated with reproduction were noted, including for the oviposition-related hormone ovipostatin and enzymes involved in metabolism of bioactive amines like dopamine or serotonin. Shedding snails exhibited responses consistent with the need for tissue repair. Both generalized stress and immune factors immune factors (VIgLs, PGRPs, BGBPs, complement C1q-like, chitinases) exhibited complex transcriptional responses in this compatible host-parasite system.

**Significance:**

This study provides for the first time a large sequence data set to help in interpreting the important vector role of the neglected snail *B*. *pfeifferi* in transmission of *S*. *mansoni*, including with an emphasis on more natural, field-derived specimens. We have identified *B*. *pfeifferi* targets particularly responsive during infection that enable further dissection of the functional role of these candidate molecules.

## Author summary

*Biomphalaria pfeifferi* is the world’s most important snail vector for the widespread human-infecting blood fluke *Schistosoma mansoni*. Despite this, we know relatively little about the biology of this highly compatible African snail host of *S*. *mansoni*, especially for specimens from the field. Using an Illumina-based dual-seq approach, we captured a portrait of the transcriptional responses of Kenyan snails that were either uninfected with *S*. *mansoni*, or that harbored 1-day, 3-day, or cercariae-producing infections. Responses to infection were influenced both by the extent of schistosome gene expression and infection duration. We note and discuss several alterations in transcriptional activity in immune, stress and reproduction related genes in infected snails and the *B*. *pfeifferi* symbionts detected. Several host genes were highly up-regulated following infection and these might comprise excellent candidates for disruption to diminish compatibility. This study provides for the first time a large sequence dataset to help in interpreting the important vector role of *B*. *pfeifferi* in transmission of *S*. *mansoni*, including with an emphasis on more natural, field-derived specimens.

## Introduction

Schistosomiasis is one of the world’s most prevalent neglected tropical diseases with over 218 million people worldwide requiring preventive chemotherapy in 2015, 92% of those occurring in 41 countries in Africa [1]. Human schistosomiasis has a greater public health impact than usually appreciated [2], often with a disproportionate impact on children, in whom it can cause both cognitive and physical impairments [3–6]. There is a growing consensus that we need to supplement chemotherapy with other control methods, including control of the obligatory molluscan intermediate host of schistosomes [7–10]. Snail control has been identified as an important component of the most successful control programs [11].

Among the most important schistosome species infecting humans and the one with the broadest geographical range is *Schistosoma mansoni*. *Biomphalaria pfeifferi* is one of 18 *Biomphalaria* species known to transmit *S*. *mansoni*. *Biomphalaria pfeifferi* has a broad geographic distribution in sub-Saharan Africa where the majority of cases of *S*. *mansoni* occur and exhibits a high degree of susceptibility to *S*. *mansoni* [12–16]. For instance, *B*. *pfeifferi* typically shows high infection rates (50%+) following exposure to *S*. *mansoni* from locations throughout Africa, but even to isolates originating from the Americas [12]. For these reasons, it can be argued that *B*. *pfeifferi* is the world’s most important intermediate host for *S*. *mansoni*. Understanding the role of *B*. *pfeifferi* in human schistosomiasis transmission becomes more critical because expanding agriculture and water development schemes [17] and climate change [18,19] threaten to alter the geographic range of both this snail species and of *S*. *mansoni* as well.

Given *B*. *pfeifferi*’s importance in transmission of *S*. *mansoni*, it is surprising we lack even the most basic information at the molecular level about its interactions with, and responses to, *S*. *mansoni*. Such responses could be particularly interesting in the case of *B*. *pfeifferi* because it differs from other major *S*. *mansoni*-transmitting snail species in that it is a strong preferential selfing species, a characteristic potentially resulting in low genetic diversity within populations [20–23]. Our relative ignorance regarding *B*. *pfeifferi* reflects the simple fact that it is often difficult to maintain this species in the laboratory, in contrast to the Neotropical snail *B*. *glabrata* which has been the standard model laboratory snail host for *S*. *mansoni* for decades [24]. *Biomphalaria glabrata* surely remains an important intermediate host of *S*. *mansoni* in the Neotropics, but given that the vast majority of *S*. *mansoni* cases occur in sub-Saharan Africa, it is critical that we extend more attention to the relevant African snail, *B*. *pfeifferi*.

The advent of genomics approaches including high throughput sequencing techniques have lead over the past decade to several studies of *Biomphalaria* snails and their interactions with *S*. *mansoni* and other trematodes including echinostomes. All of these studies have been undertaken with *B*. *glabrata* and have been amply reviewed and discussed [25–36]. In addition, the report of the international consortium on the *Biomphalaria glabrata* genome has now been published [37]. Ironically, the African *Biomphalaria* species that are responsible for transmitting the most *S*. *mansoni* infections by far have been largely ignored with respect to application of modern high-throughput sequence-based tools.

Projects going beyond the study of individual genes or gene families of *B*. *glabrata* began with studies of expressed sequence tags [38–40], ORESTES studies [41,42], and then microarrays [43,44]. These studies showed *B*. *glabrata* has the capacity for more diverse immune responsiveness than previously known, including production of diversified molecules like FREPs (fibrinogen-related proteins) [28,45,46]. Hanington *et al*. [47] examined the transcriptional responses of *B*. *glabrata* during the intramolluscan development of both *S*. *mansoni* and *Echinostoma paraensei*, and showed snail defense-related transcripts were generally down-regulated starting shortly after infection. A later generation array including ~31,000 ESTs from *B*. *glabrata* provided new insights into how the APO or amebocyte-producing organ of *B*. *glabrata* responds to immune challenge [48], and to the effects on *B*. *glabrata* transcriptional responses of the molluscicide niclosamide that is commonly used for snail control operations [49].

Additional recent studies of the interactions between *B*. *glabrata* and *S*. *mansoni* have focused on genetic linkage studies to identify chromosome regions of interest that contain genes influencing resistance to infection [32,50,51]. Functional studies have also used RNAi to knock-down particular *B*. *glabrata* gene products shown to influence susceptibility to *S*. *mansoni* [30–32,52].

Relevant to the present study, Deleury *et al*. [53] published the first Illumina sequencing study with *B*. *glabrata*, and identified 1,685 genes that exhibited differential expression after immune challenge. More recent studies employing RNA-Seq have identified *B*. *glabrata* genes associated with a state of heightened innate immunity [54] or with differential response of FREPs in *B*. *glabrata* strains differing in their susceptibility to *S*. *mansoni* [34]. Despite the fairly extensive efforts with respect to gene and genomic sequencing, gene profiling, or transcriptomics for *B*. *glabrata* and to a lesser extent for *Oncomelania hupensis* [55,56], the snail host of *Schistosoma japonicum*, to date there have been no equivalent studies published for *B*. *pfeifferi*, or for other schistosome-transmitting planorbid snails, including species of *Bulinus*, several of which transmit members of the *Schistosoma haematobium* species group in Africa, southern Europe and southwest Asia.

With this in mind, we have undertaken an Illumina RNA-Seq study of *B*. *pfeifferi*, and of *B*. *pfeifferi* infected with *S*. *mansoni* for 1 or 3 days, or with naturally acquired cercariae-shedding or “patent” infections. The intramolluscan transcriptional responses of *S*. *mansoni* will be the subject of a separate paper. The challenge of parsing *S*. *mansoni* sequences from the aggregate of reads obtained from infected *B*. *pfeifferi* has been aided by availability of the *S*. *mansoni* genome [57] and stage-specific transcriptional studies for *S*. *mansoni* [58–60].

Our view of schistosome-snail encounters has also been largely formed by studies of lab-reared snails and schistosomes. RNA-Seq offers a way to bridge and expand upon these traditional views by revealing the detailed molecular and cellular mechanisms taking place in genetically diverse hosts and parasites. This is the first Illumina study performed on samples of both field-derived vector snails and their corresponding schistosome parasites, adding a unique perspective to our understanding of schistosome transmission “in the wild” in endemic regions. This approach also serves to remind us that the snails targeted for infection by schistosome miracidia in the field are best considered as holobionts with potentially complex sets of symbiotic associates [61,62]. Finally, we note that this study will add to the literature a considerable amount of new data for *B*. *pfeifferi*, an important neglected vector species that has hitherto been understudied. Included among the snail genes highlighted are several that relate to stress, immune or reproductive functions, or that may be key players in influencing the noteworthy widespread ability of this snail to support schistosomiasis transmission.

## Methods

### Ethics and permissions statements

We enrolled human subjects who provided fecal samples containing *Schistosoma mansoni* eggs that were hatched to obtain miracidia used to infect some of the *Biomphalaria pfeifferi* snails used in this study. Fecal samples were obtained and pooled from five *S*. *mansoni*-positive primary school children aged 6–12 years from Obuon primary school in Asao, Nyakach area, Nyanza Province, western Kenya (00°19’01”S, 035°00’22”E). Written and signed consent was given by parents/guardians for all children. The KEMRI Ethics Review Committee (SSC No. 2373) and the UNM Institution Review Board (IRB 821021–1) approved all aspects of this project involving human subjects. All children found positive for *S*. *mansoni* were treated with praziquantel following standard protocols. Details of recruitment and participation of human subjects for fecal collection are described in Mutuku *et al*. [15]. This project was undertaken with approval of Kenya’s National Commission for Science, Technology, and Innovation (permit number NACOSTI/P/15/9609/4270), National Environment Management Authority (NEMA/AGR/46/2014) and an export permit has been granted by the Kenya Wildlife Service (0004754).

### Sample collection and experimental exposures

*Biomphalaria pfeifferi* used in Illumina sequencing were collected from Kasabong stream in Asembo Village, Nyanza Province, western Kenya (34.42037°E, 0.15869°S) in November 2013. Snails were transferred to our field lab at The Centre for Global Health Research (CGHR) at Kisian, western Kenya. Snails sized 6-9mm in shell diameter were placed into 24-well culture plates and exposed to natural light to check for the shedding of digenetic trematode cercariae, including cercariae of *S*. *mansoni* [15]. Snails found to be shedding cercariae of other digenetic trematode species were excluded from this study.

Snails shedding *S*. *mansoni* cercariae and non-shedding snails (controls) were separated and held for one day in aerated aquaria containing dechlorinated tap water and boiled leaf lettuce. After cleaning shells with 70% EtOH, whole shedding and control snails were placed individually into 1.5ml tubes with 1ml of TRIzol (Invitrogen, Carlsbad CA) and stored at -80°C until extraction.

*Biomphalaria pfeifferi* confirmed to be uninfected were exposed to *S*. *mansoni* using standard methods to hatch the parasite eggs [15]. Snails were individually exposed to 20 miracidia for 6 hours in 24-well culture plates and then returned to aquaria. At 1 and 3 days post-infection (d), snails were collected and stored in TRIzol as described above. We chose not to maintain the field-derived snails for longer intervals post-infection as we did not want them to lose their unique field-associated properties while maintained in laboratory aquaria.

In addition to the Illumina RNA-Seq samples indicated above and mentioned throughout this study, we have RNA-Seq data from *B*. *pfeifferi* obtained from two 454 GS FLX (Roche, Basel Switzerland) runs and six Illumina-sequenced *B*. *pfeifferi* exposed to molluscicide, all field-derived from Kenya (Table 1). These reads were used to aid assembly of the *B*. *pfeifferi de novo* transcriptome and were not included in expression studies.

**Table 1:** Samples used for the study with total read numbers and the percent of reads mapping to the *S*. *mansoni* genome that were filtered prior to *de novo* assemblies.

Field-collected samples	Replicate	Abbreviation	Paired-end reads mapping to *S*. *mansoni* genome‡	Paired-End Reads/Sample (post- quality filtering)
*B*. *pfeifferi* control	1	control-R1	0.07%	28,903,992
	2	control-R2	0.08%	34,318,971
	3	control-R3	0.04%	27,557,936
*B*. *pfeifferi* x *S*. *mansoni* 1 day post infection (1d)	1	1d-R1	0.1%	36,450,649
	2	1d-R2	1.5%	33,634,117
	3	1d-R3	1.9%	30,932,207
*B*. *pfeifferi* x *S*. *mansoni* 3 days post infection (3d)	1	3d-R1	4.1%	30,648,913
	2	3d-R2	0.1%	26,445,297
	3	3d-R3	13.2%	31,159,822
*B*. *pfeifferi* shedding *S*. *mansoni* (S)	1	shedding-R1	3.7%	32,200,842
	2	shedding-R2	8.2%	33,570,583
	3	shedding-R3	0.5%	27,569,638
*B*. *pfeifferi* control x molluscicide	1	*	*	35,289,769
	2	*	*	34,450,509
	3	*	*	25,652,418
*B*. *pfeifferi* shedding *S*. *mansoni* x molluscicide	1	*	*	30,587,208
	2	*	*	35,071,339
	3	*	*	28,843,961

*Samples used in the assembly but expression results not discussed in this paper

^‡^ See Methods for explanation of *S*. *mansoni* read mapping

### RNA extraction, library preparation, and sequencing

Individual snails stored in TRIzol were homogenized using plastic pestles (USA Scientific, Ocala FL). For each biological treatment (control, 1d, 3d, and shedding), total RNA was purified separately from three individual snails (each snail a biological replicate) using the TRIzol protocol provided by the manufacturer (Invitrogen, Carlsbad CA). RNA samples were further purified using the PureLink RNA Mini Kit (ThermoFisher Scientific, Waltham MA). Genomic DNA contamination was removed with RNase-free DNase I (New England BioLabs, Ipswich MA) at 37°C for 10 minutes. This combination method based on the two RNA extraction assays had been developed in our lab and proved to produce a high quality of RNA from snail samples [47]. RNA quality and quantity was evaluated on a Bioanalyzer 2100 (Agilent Technologies, Santa Clara CA) and Nanodrop 2000 (ThermoFisher Scientific, Waltham MA).

Complementary DNA (cDNA) synthesis and Illumina Hi-Seq sequencing was performed at the National Center for Genome Resources (NCGR) in Santa Fe, NM. Most liquid handling was performed by a Sciclone G3 Automated Liquid Handling Workstation (Caliper Life Sciences, Hopkinton MA) with Multi TEC Control (INHECO, Martinsried Germany). Synthesis of cDNA and library preparation was prepared using Illumina TruSeq protocol according to the manufacturer’s instructions (Illumina, Carlsbad CA). Complementary DNA libraries were paired-end sequenced (2x 50 base reads) on a HiSeq2000 instrument (Illumina, Carlsbad CA).

### Pre-processing of Illumina reads and isolation of *B*. *pfeifferi* reads

Sequencing adapters, nucleotides with a Phred quality score <20 within a sliding window of 4bp, and non-complex reads were removed using Trimmomatic v.0.3 [63]. Raw read quality control checks were performed before and after Trimmomatic filtering using FastQC (http://www.bioinformatics.babraham.ac.uk/projects/fastqc/).

**Fig 1:**
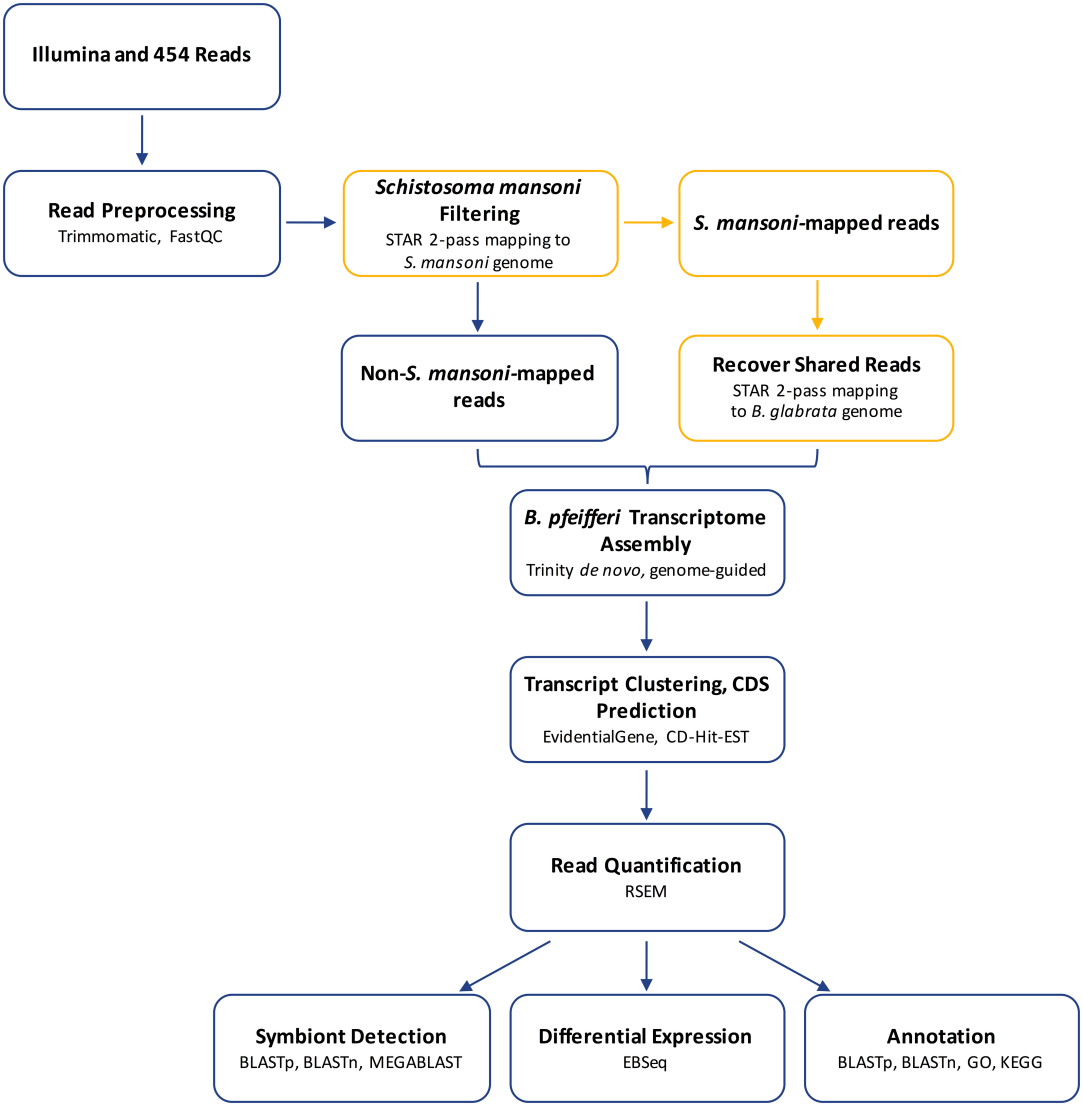
Overview of novel bioinformatics pipeline developed to isolate and analyze *B*. *pfeifferi* transcriptomic expression from dual RNA-Seq data.

To reduce assembly of chimeric transcripts, we created a novel pipeline to separate reads of related organisms when only one organism has a sequenced genome while also allowing for recovery of shared reads (Fig 1). First, all reads (including control samples) that passed quality filtering were aligned to the *S*. *mansoni* genome (GeneDB: *S*. *mansoni* v5.0) using STAR v.2.5 2-pass method [64] or Tophat v.2 [65] (see Table 1 for alignment percentages). From examination of the percentage values in Table 1, it may be interpreted that unexposed control actually harbor *S*. *mansoni*. However, the reads contributing to the positive percentage values for the controls are ones that we have found to be shared with either *B*. *glabrata* or another organism such that they represent a background level of sequence similarity obtained by chance. Although partial mapping of reads may occur, none appear to be expressed *S*. *mansoni* transcripts. None of the unexposed control reads mapping to the *S*. *mansoni* genome are unequivocally *S*. *mansoni*. By contrast, *S*. *mansoni*-exposed snails (1d, 3d, shedding) all expressed bona fide *S*. *mansoni* genes. Only in 1d, 3d, and shedding snails were transcripts clearly distinctive to *S*. *mansoni* found, such as venom allergen proteins (SmVal) (Accessions: AAY43182.1, AAY28955.1, AAZ04924.1, ABO09814.2), tegument allergen-like proteins (Accession: P14202), and cercarial stage-specific proteins (Accession: ABS87642.1), verifying the presence of a *S*. *mansoni* infection. This explanation also serves to verify that individual snails (such as 1dR2) with low *S*. *mansoni* percentages were indeed infected, such that they could be expected to be responsive to infection. Therefore, relatively low *S*. *mansoni* genome mapping, especially for shedding-R3, should not be interpreted that the infection was not successful, but rather as an indication of the transcriptional activity.

Reads that mapped to *S*. *mansoni* were also cross-examined by mapping to the version BglaB1 of the *B*. *glabrata* genome (https://www.vectorbase.org/organisms/biomphalaria-glabrata) using STAR. Reads that first mapped to *S*. *mansoni* and then also to *B*. *glabrata* were determined to be shared reads and added to the reads destined for *B*. *pfeifferi* transcriptome *de novo* assembly.

One issue encountered was to deal with both paired- and single-end reads resulting from initial quality filtering and from discordant or single-mate mapping to the *S*. *mansoni* genome. Pseudo-mate reads were created to allow maximum read usage in all stages of analysis (details and script available at https://github.com/lijingbu/RNA-Seq-Tools). This tool, pseudoFastqMate.pl, creates pseudo mate reads for single reads in a fastq file by generating a string of N’s the same length and quality score as its mate read. Reads entirely made up with Ns were ignored during the mapping process and have no impact on the final alignment and read counts.

### *De novo* transcriptome assembly and annotation

Unaligned paired and unpaired reads, determined not to solely belong to *S*. *mansoni*, were assembled using Trinity v2.2 RNA-Seq *de novo* assembler [66,67]. Trinity *de novo* and *B*. *glabrata* genome-guided assemblies were employed to maximize the chances of recovering unique *B*. *pfeifferi* transcripts. The *de novo* assemblies were concatenated and redundancy reduced using the EvidentialGene tr2aacds pipeline [68]. EvidentialGene determines the best set of transcripts based on the coding potential of transcripts generated from multiple assemblies. Only primary transcripts, denoted in EvidentialGene as “okay” and “okalt” were used in further analysis. *In silico* translation of the transcriptome was done using TransDecoder v3.0 (https://transdecoder.github.io) [65] to extract long open reading frames (ORFs) and identify ORFs with homology to known proteins with blast and pfam searches.

*Biomphalaria pfeifferi* CDS were annotated based on their closest homologs and predicted functional domains in the following databases and tools: BLASTp with NCBI non-redundant protein database (sequence identity >30%, E-value <10^−06^), BLASTn with NCBI nucleotide database (sequence identity >70%, E-value < 10^−06^), Gene Ontology [69], KEGG [70], and InterProScan5 [71]. For query CDS whose top hit was “uncharacterized”, “hypothetical”, or otherwise unknown, the consensus hit (of up to 20 hits that also meet minimum sequence identity and E-value requirements shown above is reported to help elucidate any putative function. Additionally, *B*. *pfeifferi* CDS were further scrutinized against molluscan transcripts and proteins identified in the literature.

### Identification of non-snail and non-parasite reads

As a consequence of sequencing field-collected specimens, we expected some reads to be of non-*B*. *pfeifferi* and non-*S*. *mansoni* origin. Screening for the presence of third party symbionts was one of our motivations for investigating field-derived snails in the first place. We performed the *de novo* assembly pipeline without first removing non-snail or non-schistosome sequences to get a more complete view of the complex environment in which *S*. *mansoni* development takes place. CDS coverage, sequence identity, and E-value of BLASTn, BLASTp, and MEGABLAST results were all taken into consideration when determining organism identification. The BLASTn and MEGABLAST against the NCBI nucleotide database had minimum sequence identity of 70% and E-value <10^−06^ and the BLASTp against the NCBI protein database had a minimum sequence identity of 30% and E-value <10^−06^. Query coverage (qcov) was also calculated in all BLASTs. When different BLASTs disagreed in their taxonomic assignment, the hit with highest percent query coverage, highest sequence identity, and lowest E-value was chosen, in that order. Although minimum parameters were set, nearly all CDS BLAST hits exceeded these bounds. BLASTp hits tended to have better quality hits because nucleotide sequences from the NCBI nucleotide database often contained non-coding regions that our CDS lack. CDS designated as “undetermined” had hits that did not meet minimum BLAST parameters. CDS that had a non-molluscan BLAST hit but still mapped to the *B*. *glabrata* genome (sequence identity >70%, E-value <10^−06^) were considered “shared” sequences.

Non-*B*. *pfeifferi* and non-*S*. *mansoni* CDS were categorized into 14 broad taxonomic groups: Mollusca, Amoebozoa, SAR, Viruses, Plantae, Fungi, Bacteria, Rotifera, Platyhelminthes, Arthropoda, Annelida, Nematoda, Chordata, and Miscellaneous. Potential trematode CDS were further filtered to require a minimum of 70% query coverage. Genomes and CDS of specific symbionts of interest (if publicly available) were interrogated using BLASTn (>70% identity, E-value <10^−06^, query coverage >70%).

### Identification of toll-like receptors (TLR) and variable immunoglobulin lectins (VIgLs)

Given that a number of previous studies of *Biomphalaria* immunobiology have focused on molecules with TLR or immunoglobulin domains, we undertook an analysis of these groups of molecules. *Biomphalaria pfeifferi* CDS with a BLASTp or BLASTn annotation as a toll-like receptor (TLR), were further screened for toll/interleukin-1 receptor (TIR), leucine-rich repeats (LRR), and transmembrane regions with InterProScan5 and TMHMM (Transmembrane helix prediction based on hidden Markov model) [72]. CDS identified as complete TLRs contained TIR, transmembrane, and LRR domains. Similarly, CDS annotated as a VIgL (FREPs, CREPs, GREPs, and FREDs) were scanned for an immunoglobulin domain and a fibrinogen, C-type lectin, or galectin domain using InterProScan5. For CDS to be identified as a FREP, CREP, or GREP, they had to contain a lectin domain and at least one immunoglobulin domain.

### Transcriptome completeness

To estimate the completeness of our *B*. *pfeifferi* transcriptome assembly and assess similar transcripts across related species, *B*. *pfeifferi* predicted ORFs were compared to other molluscan peptides (the cephalopod *Octopus bimaculoides*, the oysters *Crassostrea gigas* and *Pinctada fucata*, the owl limpet *Lottia gigantea*, the California sea hare *A*. *californica*, as well as two pulmonates: *B*. *glabrata* and *Radix balthica*) using BLASTp (sequence identity >30%, E-value <10^−06^). ORFs with 100 or more amino acids were extracted from each transcriptome. To maximize sensitivity for retaining ORFs that may have functional significance, predicted ORFs were scanned for homology to known proteins in the Uniref90 database with a subsequent search using PFAM and hmmer3 to identify protein domains.

### Differential expression analyses

Properly paired reads not filtered as *S*. *mansoni* were mapped to EvidentialGene-generated *B*. *pfeifferi* CDS with Bowtie2 [73]. Read abundance was quantified with RSEM (RNA-Seq by expectation maximization) [74]. Pairwise analyses for comparisons between control group and other infected groups were run in EBSeq [75]. Transcripts with a posterior probability of differential expression (PPDE) > = 0.95 were considered significant. With the aim of detecting less abundant transcripts that may still have significant biologically effects (i.e. neuropeptides), we deliberately did not set a minimum read count threshold for detection of DE CDS in EBSeq.

### Variation among infected snails with respect to representation of *S*. *mansoni* reads, and testing among them for associated differences in host responses

As noted above, field-collected specimens of both snails and schistosomes are naturally more genetically diverse than lab-reared counterparts, so variation in response among infected snails might be expected. In fact, by chance, for each of the time points studied, one of the 3 infected snails examined differed notably from the other two in having fewer normalized *S*. *mansoni* read counts (suggestive of less extensive parasite activity and/or more effective host limitation of parasite development). We hypothesized that the snail response is influenced by the extent of *S*. *mansoni* representation, as assessed by examining normalized parasite read counts from each infected snail. In addition to doing “3 controls vs. 3 infected” (3v3) comparisons, for each time point we also examined “3 control vs. 2 infected” (3v2) comparisons where the two snails harbored higher *S*. *mansoni* read counts to identify CDS whose responses were associated with *S*. *mansoni* abundance. We also performed “3 control vs. 1 infected” (3v1) comparisons where the one infected snail was the one with low *S*. *mansoni* read counts. The overall DE results include all CDS that were differentially expressed in any of the three comparisons, the results for each comparison being separately singled out and enumerated.

### Quantitative PCR validation of differential expression

cDNA was synthesized from 5μg of total RNA from the original samples by the SuperScript II First-Strand Synthesis Kit for RT-PCR (Invitrogen) in a 20μl reaction using random hexamers. Manufacturer directions were followed for the reaction profile. An additional 80μl of molecular grade water was added to the cDNA for a final volume of 100μl. qPCR target primer sequences were generated in Primer3 software [76] and details are shown in S1 Table. We tested probes for single-copy genes only and final selection of qPCR targets were chosen to highlight the variability between replicates. Primer testing verified one product was produced in traditional PCR amplification and in melt curve analyses. RT-qPCR reactions were performed in 20μl reactions according to manufacturer’s directions using SsoAdvanced Universal SYBR Green Supermix (Bio-Rad Laboratories, Hercules CA) with 0.5μM primer concentration and 2μl cDNA. Reactions were denatured at 95°C for 2 minutes followed by 40 cycles of 95°C for 5 seconds and annealing/extension and plate read for 30 seconds. Melt curve analysis was performed from 65–95°C at 0.5°C increments for 5 seconds. All biological replicates were run in technical triplicate for each transcript on a Bio-Rad CFX96 system and analyzed with Bio-Rad CFX Manager software.

## Results

### Transcriptome sequencing, assembly, and annotation

To investigate the gene expression profiles of *B*. *pfeifferi* following infection with *S*. *mansoni*, we analyzed the transcriptome from Illumina sequencing of infected snails at 1-day (1d), 3-day (3d), and from shedding snails using three biological replicates each (Table 1). The raw and assembled sequence data are available at NCBI under BioProject ID PRJNA383396. The results and statistics describing the *B*. *pfeifferi* assembly are summarized in Table 2. Trinity *de novo* transcript assemblies and additional reads from two 454 runs resulted in 1,856,831 contigs. The EvidentialGene program generated a non-redundant *B*. *pfeifferi* transcriptome of 194,344 protein-coding sequences (CDS) that includes isoforms. From nucleotide sequence length histograms, we calculated that more than half of the CDS were between 300–499 nucleotides with 6.7% > = 1500 nucleotides (S1 Fig).

**Table 2:** Illumina sequencing and *B*. *pfeifferi de novo* transcriptome assembly summary metrics.

**Raw Illumina data**	
Number of paired-end reads sequenced	563,288,171
Number of reads sequenced	1,126,576,342
Reads surviving quality filtering and trimming	1,120,661,048
Reads surviving *S*. *mansoni* filtering	1,048,936,142
**Filtered reads used in *de novo* assemblies**	**1,048,936,142**
**Assembled contigs**	**1,805,496**
Trinity *de novo* Illumina	201,573
Trinity *de novo* Illumina including shared reads	225,929
Genome-guided Trinity *de novo* Illumina	62,682
Genome-guided Trinity *de novo* 454	71,199
Additional 454 reads	1,244,113
**EvidentialGene clustering**	
Okay + Okay alternate coding sequences (CDS)	**194,344**
% GC	44.24
N_50_	654
Longest CDS length	28,302
Median CDS length	447
Average CDS length	634.57
Clusters > = 1Kb	24,802
% positive strand orientation	53.2%
% negative strand orientation	46.8%
**TransDecoder-predicted open reading frames (ORFs)**	
Total predicted ORFs (minimum length = 100 aa)	166,921
Longest ORF length (aa)	9,434
Median ORF length (aa)	157
Average ORF length (aa)	232.07
Average ORF size of 1,000 longest CDS	2014.1

Five publicly available databases were used to annotate and obtain functional information for the CDS (S1 File; Table 3). The top 20 most common GO assignments are shown in S2 Fig. Six KEGG categories are shown with their constituent classes organized by abundance in S3 Fig. Altogether, 179,030 of 194,344 total (92.1%) CDS were annotated from at least one of the five databases shown in Table 3.

**Table 3:** CDS and predicted protein annotations using publicly available databases.

Public Database	Annotation Summary
BLASTp x nr	140,484 CDSs (72.3%) 49,518 unique protein identities
BLASTn x nt	128,028 CDSs (65.9%) 26,708 unique nt identities
InterProScan	137,778 (70.9%)
Gene Ontology (GO)	50,870 CDSs (26.2%)
Unique Molecular Function	3,246
Unique Cellular Component	1,618
Unique Biological Process	8,282
KEGG	145,197 CDSs (74.7%)
Unique KEGG orthologous groups	3,824
Unique KEGG pathways	387
Unique KEGG classes	46
Unique KEGG categories	6
Cellular Processes	13,845
Environmental Information Processing	16,093
Genetic Information Processing	13,722
Human Diseases	32,748
Metabolism	41,022
Organismal Systems	27,767

### Identification of toll-like receptors (TLRs) and variable immunoglobulin lectins (VIgLs)

**Fig 2:**
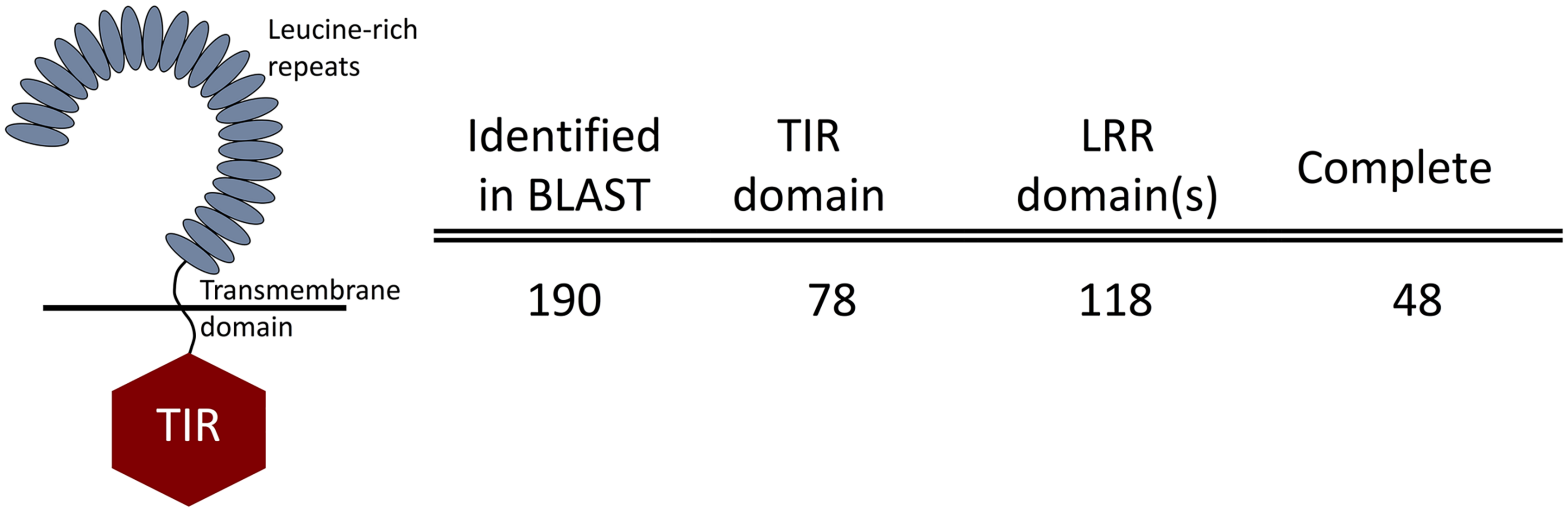
Identification of the innate immune recognition receptors TLRs in *B*. *pfeifferi*. Partial CDS counts had a BLAST hit against a known TLR but all necessary domains could not be confidently determined by InterProScan5.

Pattern recognition receptors like TLRs and VIgLs (FREPs, CREPS, and GREPs) are key components of the innate immune response and their involvement in the *B*. *glabrata* defense response has been documented [28,77]. The *B*. *glabrata* genome contains 56 TLR (toll-like receptor) genes, 27 of which encode complete TLRs [37]. Our *B*. *pfeifferi* transcriptome had 190 CDS annotated as a homolog to a *B*. *glabrata* TLR (Fig 2). Note that numbers assigned to TLRs in *B*. *glabrata* were assigned in the order they were identified and not by homology to vertebrate TLRs. The TLR numbers we refer to for *B*. *pfeifferi* match most closely the TLR with the corresponding number from *B*. *glabrata*. InterProScan5 analysis revealed 78 of *B*. *pfeifferi* TLR CDS contain a TIR (toll/interleukin receptor) domain and 118 have at least one LRR (leucine-rich repeat) domain. In total, we found 48 complete *B*. *pfeifferi* TLRs (TIR, transmembrane, LRR domains all present) and 142 partial homologs to *B*. *glabrata* TLRs (annotated as a TLR, but not all domains complete and/or confidently identified) in our transcriptional study. Others may certainly exist in the genome of *B*. *pfeifferi*.

**Fig 3:**
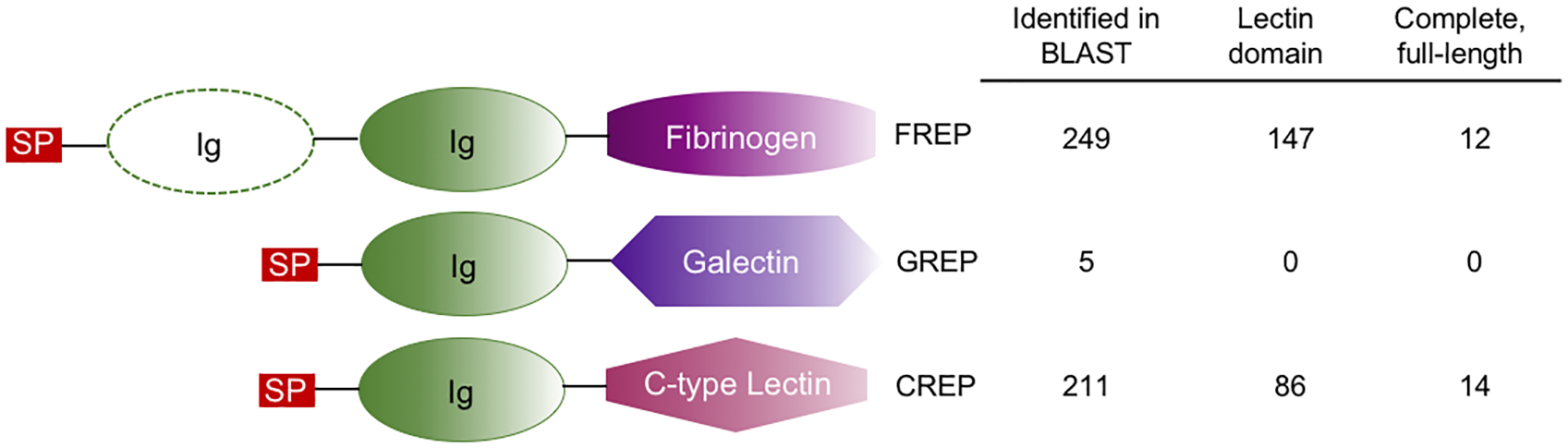
Identification of the innate immune recognition receptors VIgLs in *B*. *pfeifferi* with initial BLAST annotation and then verification of protein domains in InterProScan5.

There are 22 FREP genes in the *B*. *glabrata* genome [37,77] and all were represented in our *B*. *pfeifferi* transcriptome, at least in part. Our BLAST annotations identified 249 *B*. *pfeifferi* CDS homologous to *B*. *glabrata* FREPs and 12 of these were verified to be full-length FREP homologs (Fig 3). There were no full-length, complete GREPs identified in our transcriptome, but there were 5 CDS with a BLAST annotation homologous to one *B*. *glabrata* GREP identified by Dheilly *et al*. [77] (Fig 3). Four CREPs (C-type lectin protein) have been identified in *B*. *glabrata* [77] with 2 of the 14 full-length, complete *B*. *pfeifferi* CDS homologous to CREP 1 in *B*. *glabrata* (Fig 3).

### Sequence homology between related mollusc species

A BLASTp comparison between *B*. *pfeifferi* and *B*. *glabrata* shows high sequence similarity with 35,150 (95.8%) polypeptides shared between the two species (sequence identity >30% and E-value <1e^-06^) (Table 4). We found 1,525 *B*. *glabrata* polypeptides without homologs in our *B*. *pfeifferi* transcriptome. With respect to the 127,626 translated CDS that have homologs to *B*. *glabrata* polypeptides, more than half of these have a sequence identity greater than 90% (S4 Fig). To further assess the completeness and to enhance annotation of our *B*. *pfeifferi* transcriptome, we searched for homologous polypeptides from genomes of two additional gastropods (*Aplysia californica* and *Lottia gigantea* [78]), two bivalves (*Pinctada fucata* [79]) and *Crassostrea gigas* [80]), and one cephalopod (*Octopus bimaculoides* [81]) (Table 4). Shown in S5 Fig is one hypothesis of the phylogeny of molluscs, and mapped onto this are the mollusc genomes that are currently available [82]. Note that the percent identity of homologous sequences follows the general branching pattern. The California sea hare, *A*. *californica*, has 88.3% of its polypeptides homologous to *B*. *pfeifferi* peptides. The most distantly related mollusc, the California two-spot octopus, *O*. *bimaculoides*, is 56.7% homologous at the protein level to *B*. *pfeifferi*.

**Table 4:** Number of polypeptides queried in various molluscs and matches with *B*. *pfeifferi* TransDecoder-predicted ORFs.

Reference	# Reference polypeptides	*B*. *pfeifferi* polypeptides matched to reference polypeptides	Download location
*Biomphalaria glabrata* v1.0 [37]	36,675	127,626	https://www.ncbi.nlm.nih.gov/genome/annotation_euk/Biomphalaria_glabrata/100/
*Aplysia californica* v3.0*	27,591	99,884	http://www.ncbi.nlm.nih.gov/genome/annotation_euk/Aplysia_californica/101/
*Lottia gigantea* v1.0 [78]	188,590	74,494	http://genome.jgi.doe.gov/Lotgi1/Lotgi1.download.ftp.html
*Pinctada fucata* v2.0 [79]	31,477	77,341	http://marinegenomics.oist.jp/pearl/viewer/download?project_id=36
*Crassostrea gigas* v9 [80]	45,406	80,505	ftp://ftp.ncbi.nlm.nih.gov/genomes/Crassostrea_gigas/
*Octopus bimaculoides* v2.0 [81]	38,585	71,395	http://genome.jgi.doe.gov/pages/dynamicOrganismDownload.jsf?organism=Metazome

*Genome is publicly available at link provided

### Other organismal sequences derived from the *de novo* assembly

**Fig 4:**
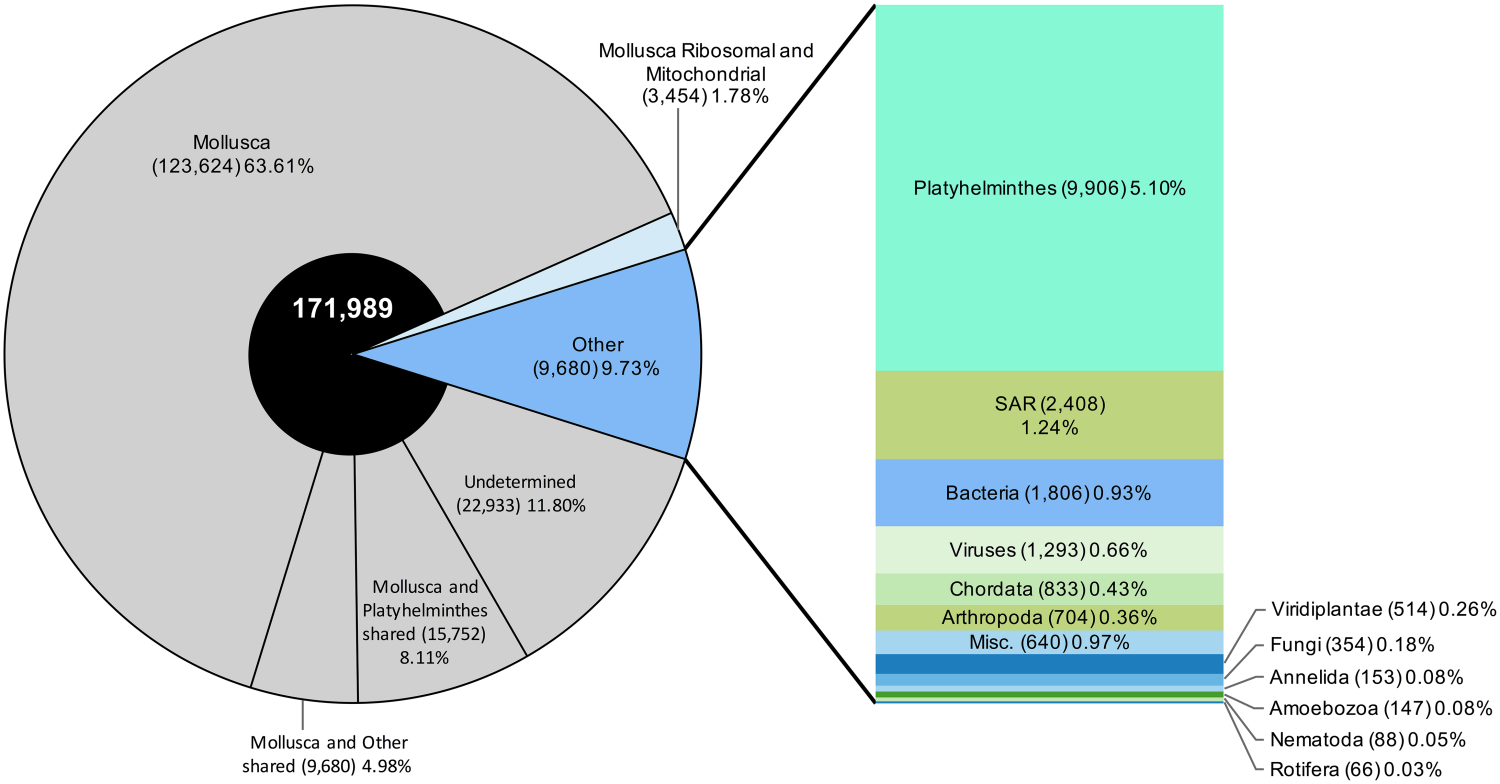
Identification of all *de novo* assembled transcripts after *S*. *mansoni* read filtering.

**Fig 5:**
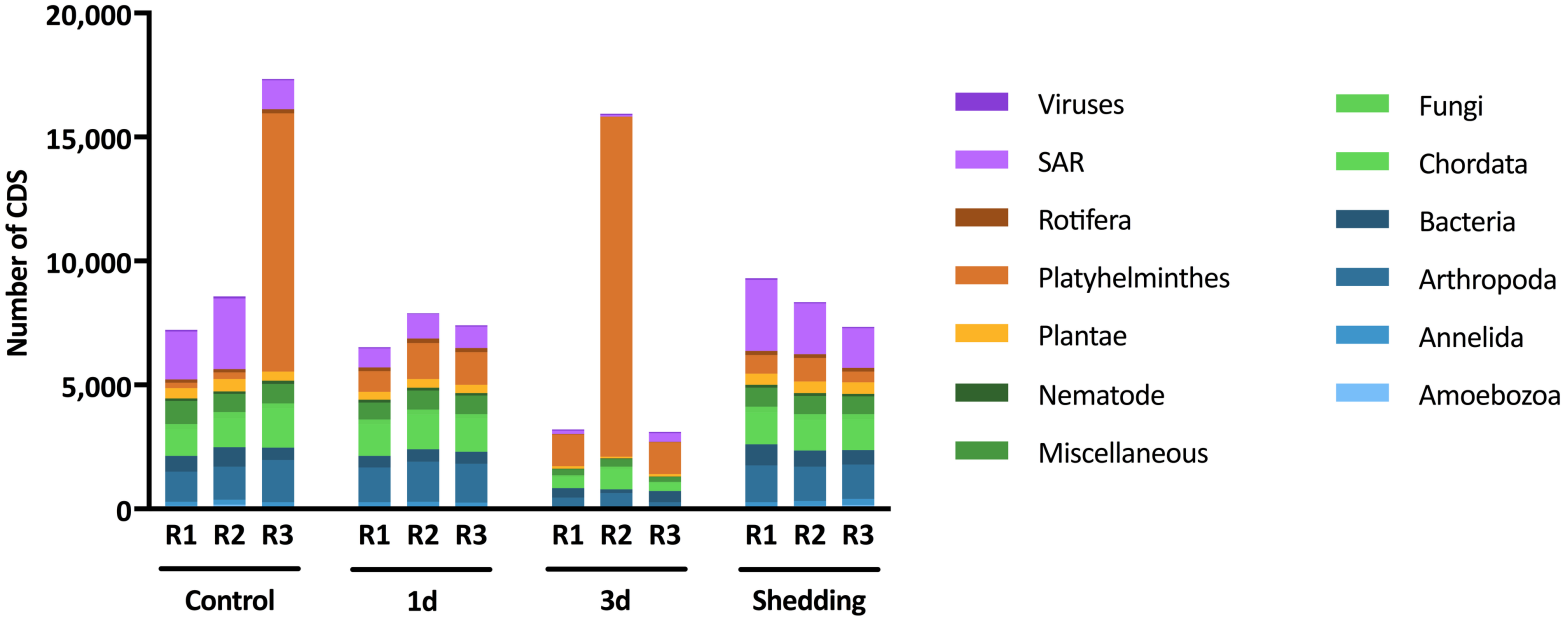
Sum of non-*B*. *pfeifferi de novo* assembled CDS for each replicate. CDS were counted as present if read count >0.

Of the 194,344 CDS assembled post-*S*. *mansoni* read filtering, 18,907 (9.73%) of these were determined to be of non-mollusc origin (Fig 4). Some of the non-*B*. *pfeifferi* transcripts found were bacteria with most belonging to the genera *Escherichia*, *Mycoplasma*, *Aeromonas*, and *Pseudomonas* (Fig 5). Among them, a CDS with homology to *Neorickettsia sp*, a known obligatory symbiont of digenetic trematodes [83], was recovered and has read counts >10 in 2 of our samples that also had relatively high counts of *S*. *mansoni* (3d-R3 and shedding-R1) (Table 1; S2 File). In addition, there are three CDS assembled from the infected 454 *B*. *pfeifferi* sample that were identified as *Paenibacillus* spp. and were similar, but not identical, to the snail pathogen *Candidatus* Paenibacillus glabratella (S2 File) [84].

Among the eukaryotic sequences retrieved from generation of the *de novo* assembly, there are some familiar snail symbionts listed in S2 and S3 Tables including 1) *Chaetogaster* annelids, 2) *Trichodina* ciliates, and 3) *Capsaspora owczarzaki* [85] and 4) microsporidians [86–89] (see also S2 File and Discussion for further comments).

In addition to prokaryotes and eukaryotes, nearly 1,300 of our assembled CDS were provisionally identified as viruses (Fig 4). Sample Control-R2 had the highest abundance of reads mapping to the viral sequences compared to the other samples, though some putative viral sequences were recovered from all 12 snails examined.

Lastly, even after the initial screening and removal of *S*. *mansoni* reads from the nine snails with known *S*. *mansoni* infections, some reads remained that were classified as platyhelminth in origin (Fig 4). Two individual snails in particular, control-R3 and 3d-R2, the latter a replicate with low *S*. *mansoni* read counts, had many platyhelminth reads (Fig 5). We sequenced a 28S rRNA gene from cDNA of control-R3 using digenean-specific primers [90] to determine if other digeneans were present in our sample. The resulting 28S sequence was identified as belonging to the genus *Ribeiroia*, members of which are known to occur in East Africa and to infect *Biomphalaria* [91]. Most of the platyhelminth CDS present in this sample were identified as “hypothetical” but CDS with the highest read abundance are involved in membrane transport and cell structural functions. For 3d-R2, *cox1* mitochondrial gene primers amplified an amphistome sequence that groups phylogenetically with an amphistome species (provisionally *Calicophoron sukari*) that uses *B*. *pfeifferi* from East Africa as a first intermediate host [92]. Like control-R3, CDS with the highest read abundance in 3d-R2 were membrane associated and structural with the addition of several myoglobins and surface glycoprotein CDS.

### Variation among infected snails with respect to the representation of *S*. *mansoni* reads, and associated responses

**Fig 6:**
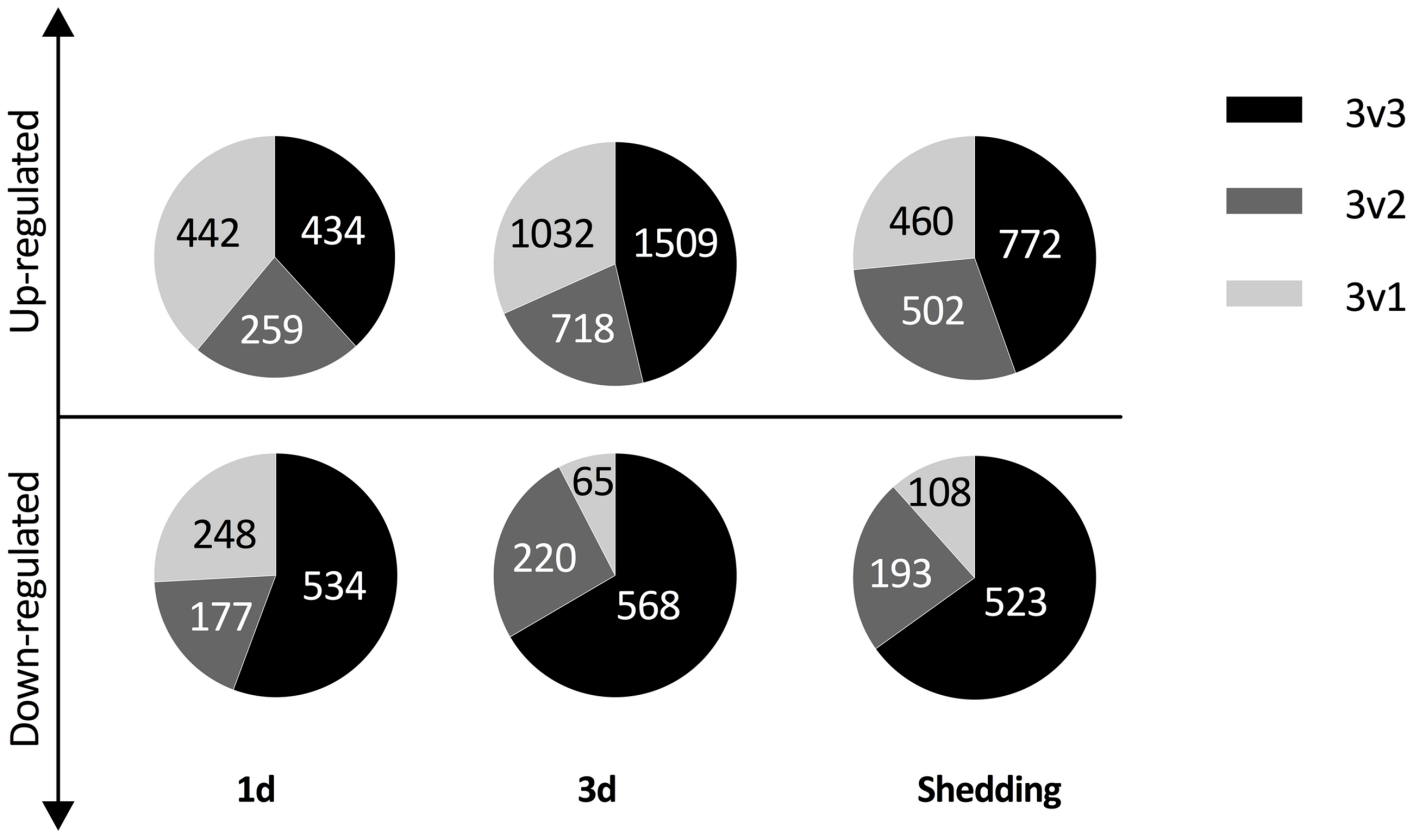
Pie charts of unique CDS found to be differentially expressed in 3v3, 3v2, and 3v1 EBSeq analyses.

The extent of representation of *S*. *mansoni* in the dual transcriptome as measured by read counts is variable among the three replicates for each development time sampled in shedding snails (Table 1). Normalized read abundance of *S*. *mansoni* housekeeping genes remained consistently high across all samples, eliminating the possibility that *S*. *mansoni* read count variability was due to sampling effects. Because of this inherent variability, we performed additional DE comparisons to the traditional 3 control v 3 experimental (3v3) replicates isolating either the two snails that contained higher *S*. *mansoni* read counts (3v2 analysis) or the one snail with the fewest *S*. *mansoni* read counts of each triplicate time point (3v1). With respect to the overall response patterns of snails that yielded either high or low numbers of *S*. *mansoni* reads, in most cases, for both up- and down-regulated CDS, the majority of significantly differentially expressed CDS fell into the 3v3 comparison category (Fig 6), indicative of uniformity of response across infected snails. For up-regulated features, there were also substantial additional numbers of significant CDS in the 3v2 or 3v1 infected categories, with the latter being greater in 2 of 3 cases. By contrast, for the down-regulated features, at 1d, the snails with high or low *S*. *mansoni* read counts did not as clearly differentiate from one another, but the snails with low read counts for *S*. *mansoni* (3v1) clearly showed an additional allotment of down-regulated features. For the other two time points, the snails with high and low *S*. *mansoni* read counts did separate from one another, and especially noteworthy is the relatively small proportion of down-regulated features in the 3v1 comparisons.

### *B*. *pfeifferi* CDS responsive during *S*. *mansoni* infection

**Fig 7:**
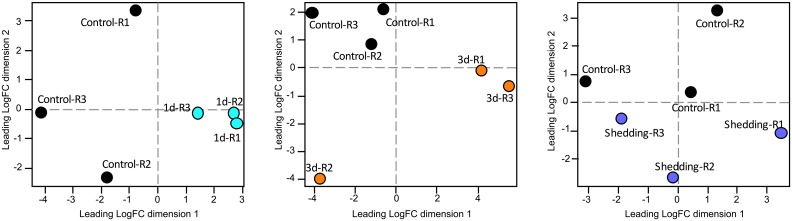
Multidimensional scaling (MDS) plots of pairwise comparisons of control versus 1d, 3d, and shedding replicates used for differential expression analyses.

**Fig 8:**
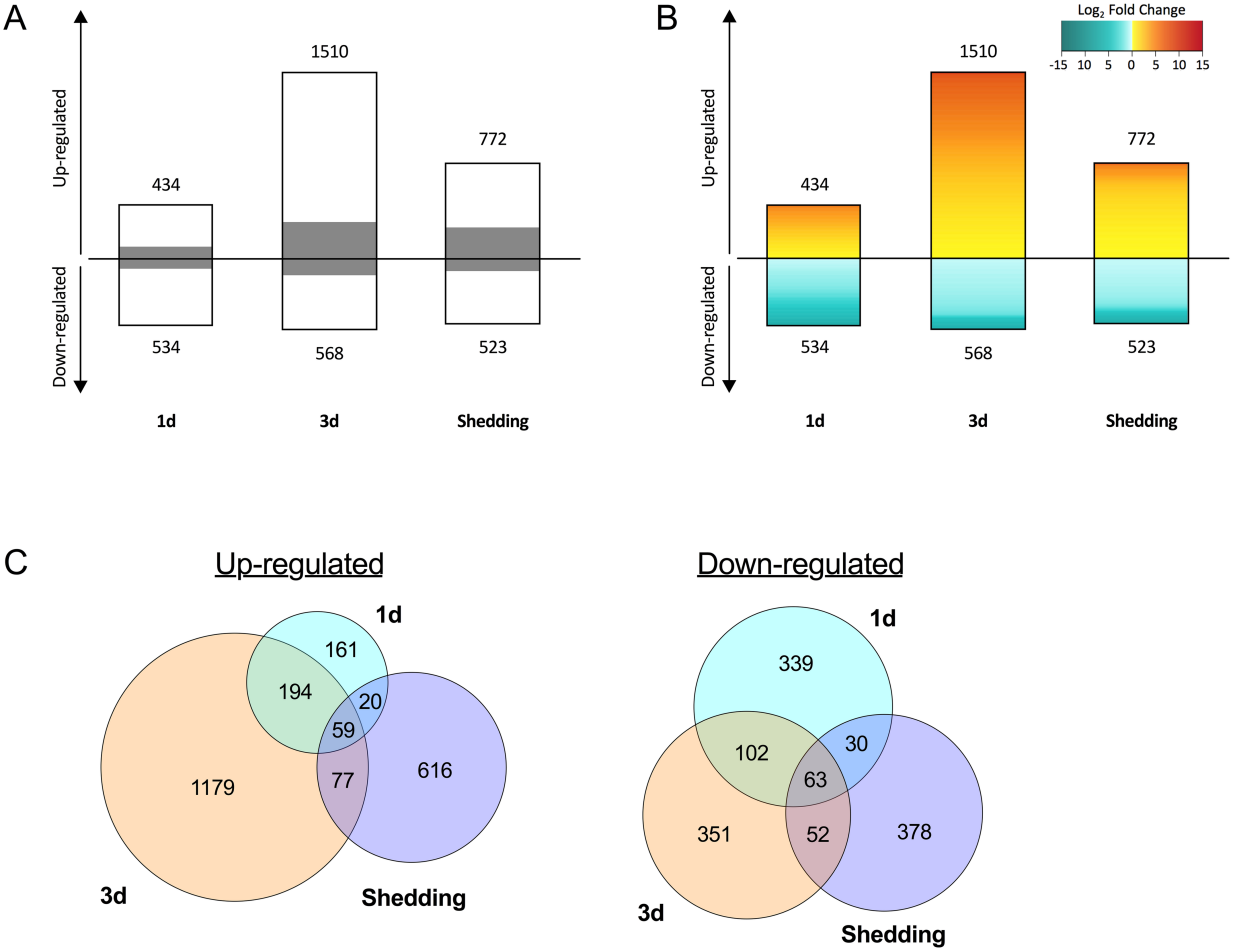
*Biomphalaria pfeifferi* differential expression profiles in 1d, 3d, and shedding snails. (A) Overall expression profiles for up- and down-regulated *B*. *pfeifferi* CDS in the 3v3 DE analysis with proportions shown for CDS with annotation known (white) and without annotation (gray) from one of the 5 databases searched (Table 3). Numbers by bars refer to numbers of up- and down-regulated features. (B) Heat map of differentially expressed *B*. *pfeifferi* CDS. (C) Up- and down-regulated *B*. *pfeifferi* CDS shared between 1d, 3d, and shedding snail groups in the 3v3 DE analysis are shown.

S3 File provides a summary of all CDS retrieved in the DE analysis, S4 File summarizes those general, reproduction or immune system features that were most differentially expressed, and Tables 5 and 6 distill CDS (see Discussion also) that we feel are most worthy of further functional study in *B*. *pfeifferi*. Multidimensional scaling (MDS) plots show that for each of the three groups of infected snails, overall transcript expression of the experimental groups is distinct from the control groups (Fig 7). At 1d, snails showed a slight preponderance of down-regulated over up-regulated CDS, but in both 3d and shedding snails, the opposite trend was observed (Fig 8A and 8B). Overall, the most transcriptional activity was in the 3d snails. All three groups of infected snails (1d, 3d, shedding) showed distinct transcriptional profiles, suggesting the snail response is different at each time point (Fig 8C). Generally, each of the three groups has more unique responsive CDS than they do in common with one another. As anticipated, 1d and 3d snails have more shared transcripts both up- and down-regulated than either do with the shedding snails.

**Table 5:** Highly up- or down-regulated *B*. *pfeifferi* CDS whose response may be required for maintaining a patent *S*. *mansoni* infection.

*B*. *pfeifferi* CDS	Annotation	Log_2_FC 3v3	Log_2_FC 3v2	Log_2_FC 3v1
evgTRINITY_DN89401_c6_g1_i1	GD13313-like	9.14	9.58	6.75
evgTRINITY_DN19832_c0_g1_i1	deleted in malignant brain tumors 1 protein-like	6.65	7.09	4.48
evgTRINITY_DN95353_c0_g1_i1	collagen alpha-3(VI) chain-like	6.41	6.80	
evglcl|G0WVJSS02FGR88	cAMP-dependent prot kinase catalytic subunit-like	6.17	6.60	4.15
evgTRINITY_DN84392_c3_g1_i1	galactocerebrosidase-like	5.41	5.53	5.14
evgTRINITY_DN104940_c0_g1_i1	cAMP-dependent prot kinase catalytic subunit-like	5.32	5.79	3.06
evgTRINITY_DN84179_c0_g1_i1	uncharacterized transporter slc-17.2-like	5.31	5.21	5.20
evgTRINITY_DN16840_c0_g1_i1	papilin-like	5.05	5.55	
evgTRINITY_GG_14665_c0_g1_i2	ctenidin-3-like	-5.43	-4.89	
evgTRINITY_DN92655_c9_g2_i1	deoxyribonuclease-1-like	-4.84	-4.63	
evgTRINITY_DN68720_c0_g1_i1	testisin-like	-4.69	-4.18	

**Table 6:** Highlights of general, reproductive, and immune responses of *B*. *pfeifferi* in response to *S*. *mansoni* infection.

**One day post-infection (1d)**			
	**General**	**Reproductive**	**Immune**
**UP-REGULATED**			
**Overall**	phospholipase A2s	Na and Cl dependent glycine transporter 2-like	dermatopontins
	endoglucanases	neuropeptide Y receptor type 5-like	ficolin-like proteins
	Proteases and protease inhibitors	DBH-like monooxygenase protein 1	macrophage man rec 1-like isoform X1
	Guanine nucleotide-binding protein-like 3		C-type lectin -6 member A-like
	Translationally-controlled tumor protein		acidic mammalian chitinase-like
			chitinase-3-like protein 1-like
			hemocytin
			laccase-15-like
			laccase-1-like
			tyrosinase-1-like
**Two snails with higher S. mansoni read counts**		FMRF-amide receptor-like	Cu, Zn superoxide dismutase
		Tyrosinase-like protein tyr-1	GTPase IMAP family members 4 and 7
			beta 1,3 glucan-bind protein-like precursor
			complement C1q-like protein
			fibrinogen-related protein 2 (FREP2)
**One snail with least S. mansoni read counts**	ATP synthase FO6		macrophage expressed gene-1
			spermine oxidase
			glutathione-S-transferase
			laccase-2-like
**DOWN-REGULATED**			
**Overall**	glyceraldehyde-3-phosphate dehydrogenase	ovipostatin 2	FREP12 and its precursors
	respiratory pigment hemoglobin	tyramine/dopamine β-hydroxylase-like	toll-like receptor 8
	insulin-like peptide 7 –modestly down	FMRF-amide isoform X2 –modestly down	cytidine deaminase
	pedal peptide 2	PTSP-like molecule	zinc metalloproteinase /disintegrin-like
	Na dependent nutrient aa transporter1-like	pheromone Alb-1	
	enterin	type 1 serotonin receptor	
	FMRF-amide isoform X2	schistosomin	
	cytidine deaminase		
	soma ferritins		
**COMPLEX (MIXED RESPONSES)**			
	collagens		
	acidic mammalian chitinase-like proteins		
	cytochrome c oxidases		
	Mucins		
	Cytochrome P450 family members		
	Multidrug resistance proteins		
	Some heat shock proteins		
**Three day post-infection (3d)**			
	**General**	**Reproductive**	**Immune**
**UP-REGULATED**			
**Overall**	phospholipase A2s	Na and Cl dependent glycine transporter 2-like	GTPase IMAPs- complex, but mostly up
	endoglucanases	temptin-like	beta-1,3-glucan binding proteins
	Proteases and protease inhibitors	kynurenine 3-monooxygenase-like	complement C1q-like proteins
	17-beta hydroxysteroid dehydrogenase type 6		probable serine carboxypeptidases (1–5)
	betaine homocysteine-methyltransferase 1-like		glutathione S-transferases
	translationally controlled tumor protein homolog		laccase-2-like
	hemoglobin type 1		
**Two snails with higher S. mansoni read counts**	insulin-related peptide-3-like	Tyrosinase-like protein tyr-1	dermatopontins
	cytochrome b	Na- and Cl-dependent taurine transporter-like	ficolins
	serine proteases alpha and beta	dopamine receptor 2-like	Cu-Zn superoxide dismutases
	ADP, ATP carrier-like protein		C-type lectin domain family 6, A-like
	heparinase-like isoform X!		chitinase-3-like protein
	serpin B6-like		chitotriosidase-1-like
			aplysianin-like proteins
			FREP2, FREP5
			macrophage-expressed gene 1 protein-like
			laccase-15-like
			tyrosinase-1-like
**One snail with least S. mansoni read counts**	profilin	ovipostatin 6	hemocytin
	cathepsin B and L1-like	yolk ferritin precursor	hemagglutinin/amoebocyte aggreg factor-like X1
	neuroglobinase-like	DBH-like monooxygenase protein 1	G-type lysozyme
	chymotrypsin-like elastase family member		sialate–O-acetylesterase-like protein
	histone transcription factor		peroxidase-like protein
			fibrinogen-like protein A
			FREP 7
			peptidoglycan-recognition proteins SC2-like
			LRR-containing 15-like, toll-like receptor 13
**DOWN-REGULATED**			
**Overall**	glyceraldehyde-3-phosphate dehydrogenase	FMRF-amide-like isoform X2 –modestly down	caveolin-1-like
	aryl hydrocarbon recep nucl translocator-like	tyramine/dopamine β-hydroxylase-like	disintegrin/metalloprot containing prot t17-like
		FMRF-amide isoform X2 –modestly down	FREP12 precursors
		PTSP-like molecule	LRR contain G-prot coupled rec 5-like
		pheromone Alb-1	alpha-crystalline B chain-like
		type 1 serotonin receptor	toll-like receptors 4 and 8
		schistosomin	cytidine deaminase
**Two snails with higher S. mansoni read counts**		tyramine/dopamine β-hydroxylase-like	
**COMPLEX (MIXED RESPONSES)**			
	ornithine decarboxylase		
	actins		
	collagens		
	tubulins		
	mucins		
	cytochrome P450 members- mostly up		
	multidrug resistance proteins		
	heat shock proteins		
	thioredoxins		
	annexins		
	putative copper-containing amine oxidases		
	soma ferritins		
**Shedding**			
	**General**	**Reproductive**	**Immune**
**UP-REGULATED**			
**Overall**	FMRF-amide receptor-like—modestly up	dopamine beta hydroxylase-like	pcrotocadherein Fat 3 or 4-like
	small cardioactive peptides	FMRF-amide receptor-like	ADAM family mig-17-like
	phospholipases A2s	ovipostatin 5	zinc metalloproteinase nas 13- & 14-like
	arginase-1-like isoform X2	DBH-like monooxygenase protein	ficolins
	reverse transcriptase		
	protease inhibitors BPTI Kunitz-domain class		
	ubiquitin ISG15		
	Angiopoietin–1 receptor		
	Angiopoietin-related 2-like		
	mucins—complex but most are up		
	soma ferritins		
**Two snails with higher S. mansoni read counts**	endonuclease G mitochondrial-like	yolk ferritin-like and snail yolk ferritin molecules	aplysianin-A-like
	zinc carboxypeptide A 1-like	Neuropeptide Y receptor type 5-like	mammal ependymin-related prot 1- like
	serpinB3-like protease inhibitor		zinc carboxypeptidase A1-like
	cystatin protease inhibitor		beta-1,3-glucan binding protein-like
	putative amine-oxidases (copper containing)		FREP 2, 7 and 14
**One snail with least S. mansoni read counts**	multiple epidermal growth factor-like domains		macrophage-expressed gene
	serine/threonine-protein kinase mos-like		C-type lectin -6 member A-like
			chitinase-3-like-protein
			LRR and Ig domain containing protein
			toll-like receptor 3
**DOWN-REGULATED**			
**Overall**	insulin-like gr fact protein acid labile subunit	ovipostatin 2	toll-like receptor 7
	pedal peptide 2	dopamine beta-hydroxylase-like	galectin-6
	profilin-like isoform X1		probable serine carboxypeptidase CPVL
	neuroglobin-like		
	calreticulin-like		
	tyrosinase tyr-3		
**Two snails with higher S. mansoni read counts**	hemoglobin		dihydropyrimidinase
	collagen-related		macrophage man recep1-like protein
			tyrosinase-3-like
**One snail with least S. mansoni read counts**			tyrosinase-1-like
**COMPLEX (MIXED RESPONSES)**			
	collagens, mixed but mostly down		
	cathepsins		
	tubulins		
	cytochromes		
	ankyrins		
	Rho GTPase-activity protein 1-like		
	cytochrome P450 family members		
	multidrug resistance proteins		
	glutathione-S-transferases		
	dermatopontins		
	GTPase IMAP family members		
	thioredoxins—but mostly up		

It should also be noted that 59 CDS were up-regulated, and 63 CDS down-regulated in common to all three groups of infected snails (Fig 8C). Those up-regulated across time points include hemocytin, CD209 antigen-like, DBH-like monooxygenase, and a fibrinolytic enzyme. Some ubiquitously down-regulated features include neural cell adhesion molecule 1-like, a TNF receptor, peroxiredoxin 5, F-box/LRR repeat protein 4-like, the cytoprotective hypoxia up-regulated protein 1-like that is triggered by oxygen deprivation and oxidative stress, glutathione-S-transferase omega-1-like, type 1 serotonin receptor 5HT-1Hel, a feeding circuit activating peptide that induces feeding behavior [93], and TLR 7.

In addition to identifying those CDS up- or down-regulated in common to all three groups of infected snails, we also identified CDS not known to be related to reproduction or defense that exhibited the highest fold expression changes in shedding snails. Snail CDS most highly up-regulated may represent molecules essential for the parasite to sustain a patent infection, or conversely, those most strongly down-regulated may otherwise interfere with parasite development in ways we do not presently understand. A selected few, that had an annotation and were consistently expressed compared to controls in each replicate, are shown in Table 5.

**Fig 9:**
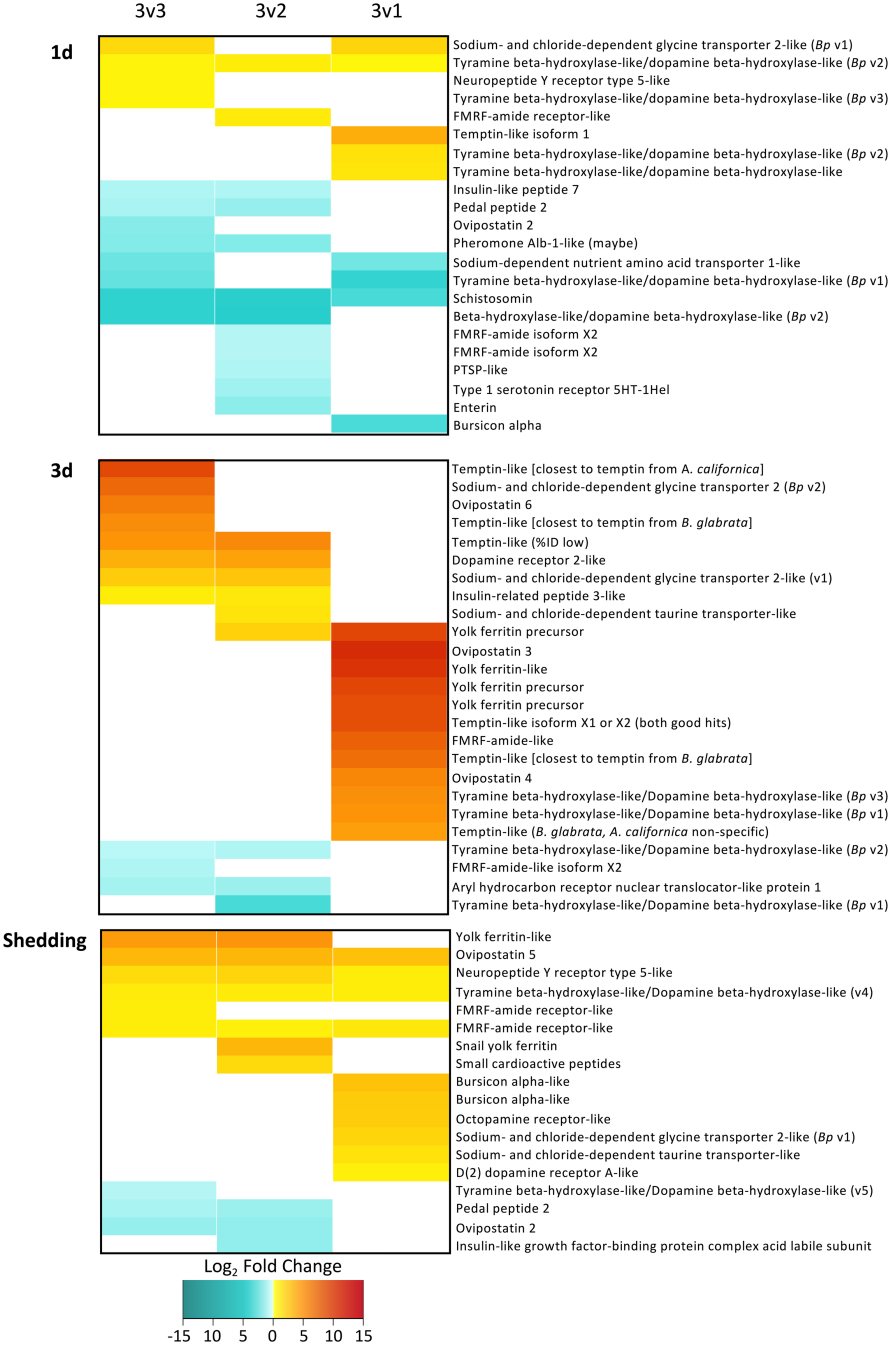
*Biomphalaria pfeifferi* CDS identified as neuropeptides, hormones, or involved in reproduction that are differentially expressed in 1d, 3d, and shedding snails. Note that the 3v3 comparison includes all 3 infected snails within a time point, whereas 3v2 includes the two infected snails with the most *S*. *mansoni* reads and the 3v1 includes only the infected snail with the fewest *S*. *mansoni* reads.

**Fig 10:**
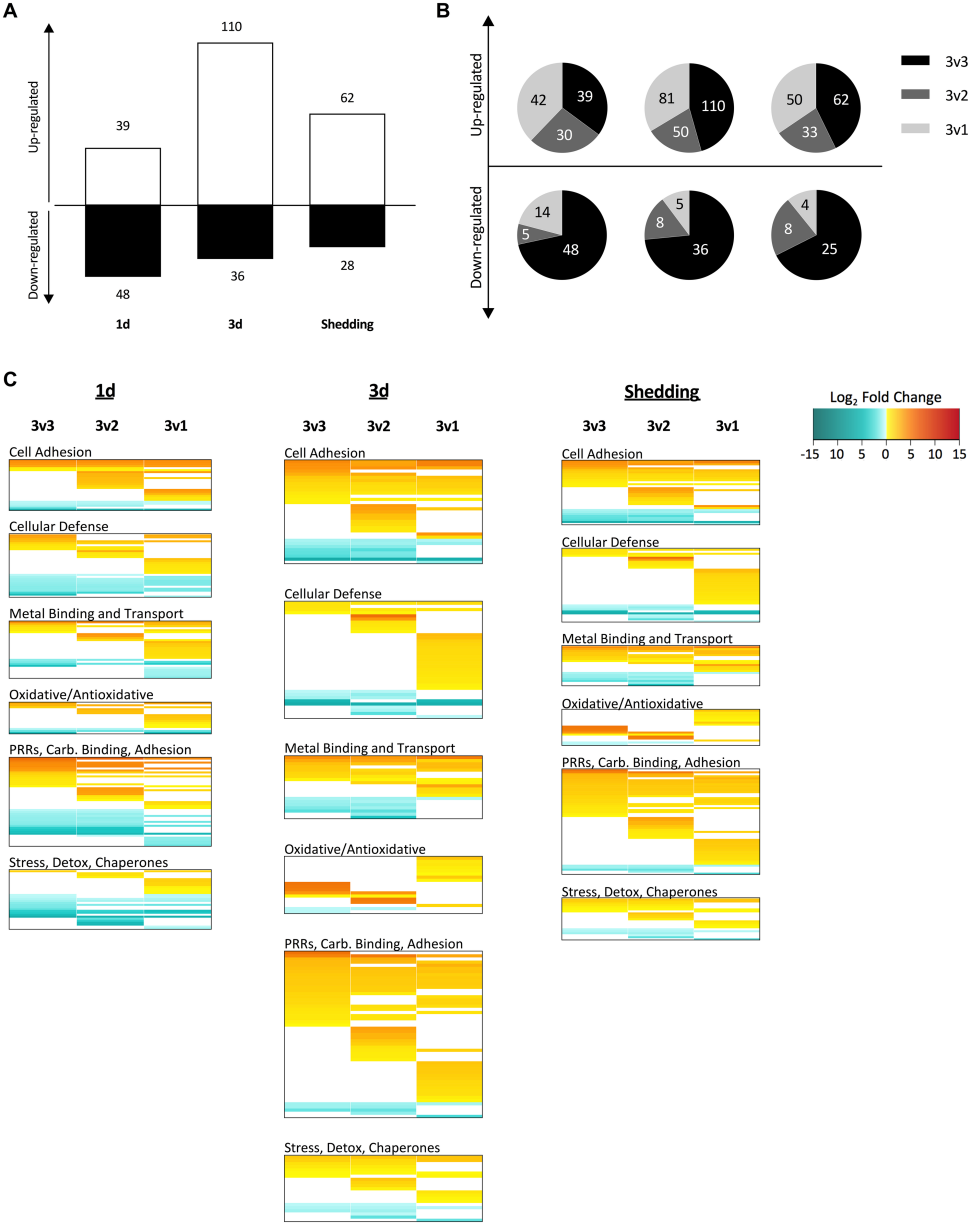
Differential expression of *Biomphalaria pfeifferi* defense-related CDS in 1d, 3d, and shedding snails. (A) Defense CDS in the 3v3 DE analysis. (B) Pie charts of proportions of CDS found to be DE in 3v3, 3v2, and 3v1 analyses. (C) Heat maps show expression levels from each of the three DE analyses highlighting the most relevant biological functional groups. Note that the 3v3 comparison includes all 3 infected snails within a time point, whereas 3v2 includes the two infected snails with the most *S*. *mansoni* reads and the 3v1 includes only the infected snail with the fewest *S*. *mansoni* reads.

With respect to transcripts involved in reproduction and potentially associated with *S*. *mansoni*-induced parasitic castration, we identified homologs to more than 100 invertebrate neuropeptides, hormones, pheromones, and polypeptides involved in reproduction, most of which have been identified in *Lymnaea stagnalis*, the sea hare *Aplysia californica*, or in *B*. *glabrata* (S4 File; Fig 9, and see Discussion). We also searched for over 500 different genes identified from previous publications that are related to immune, defense or stress responses to various pathogens or environmental stressors (S4 File; Fig 10). Each gene of interest has been organized into one of six broad functional groups for ease of interpretation, although it must be noted that many of these genes have multiple roles and could belong in several functional categories. After 1d, the majority of immune, stress and defense features were up-regulated. Noteworthy from Fig 10B is that for snails with low reads counts for *S*. *mansoni* (3v1 comparison), proportionately more features were up-regulated than for snails with high *S*. *mansoni* read counts. In two out of three comparisons, snails with low read counts for *S*. *mansoni* had fewer down-regulated genes than snails with high levels of *S*. *mansoni* read counts.

### Expression patterns validated by qPCR

**Fig 11:**
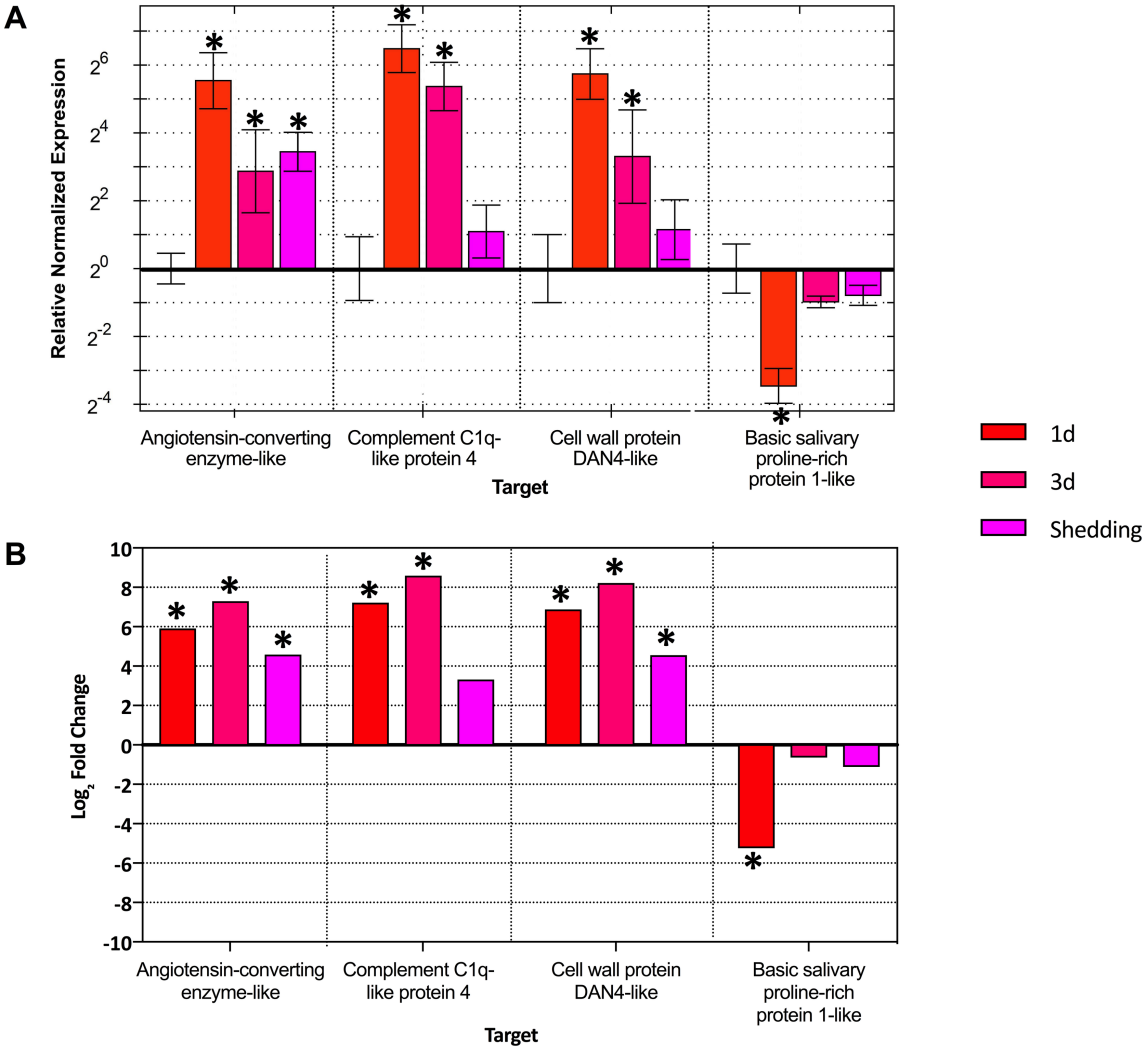
qPCR results validate Illumina RNA-Seq differential expression results. (A) Quantitative real-time PCR verifies Illumina trends among biological replicates in 1d, 3d, and shedding samples. (B) Corresponding Illumina DE results for the four genes tested. Asterisks indicate genes that are significantly DE.

Quantitative RT-PCR (qPCR) was used to validate differential expression trends by quantifying mRNA transcripts of four single-copy genes (3 up-regulated and 1 down-regulated) that highlight varying expression patterns in 1d, 3d, and shedding snails. Overall expression patterns are similar between the qPCR and Illumina DE results (Fig 11) with the same variability in DE pattern between replicates echoed in the qPCR. The only difference seen was in the gene DAN4 where the shedding group was not considered significantly DE in the qPCR analysis but was in Illumina analysis.

## Discussion

### Considerations regarding the dual-seq dataset and pipeline

This paper represents a novel pipeline for dual RNA-Seq studies where the genome of just one of the interacting partners, the parasite in this case, is available. It also highlights an advantage of using field specimens in RNA-Seq studies to reinforce the notion that individual snails are actually holobionts, and the symbiont species they carry with them may play a role in influencing susceptibility to schistosome infection or in modulating disease transmission. Also, variance among the individual snails within the groups examined presented challenges for traditional bioinformatics analyses but also revealed the heterogeneity that realistically exists among naturally diverse snails and schistosomes as they encounter one another in real-life settings in the field. We must also note that the identity and functional role for many of the CDS remain unknown thus posing rich opportunities for study for the future.

### Considerations with respect to compatibility with *S*. *mansoni*

The specific *B*. *pfeifferi-S*. *mansoni* system studied here is noteworthy for the high degree of susceptibility shown by the snail to infection [15,16]. Compatibility with *S*. *mansoni* is characteristic of *B*. *pfeifferi* throughout its range [12]. As a consequence, all snails exposed to *S*. *mansoni* or known to be shedding *S*. *mansoni* cercariae contained transcripts contributed by *S*. *mansoni*. The extent of representation of *S*. *mansoni* in the dual transcriptome is variable among the replicates for each time sampled (Table 1). Given the effects of both genetic diversity in *S*. *mansoni* [94] and in *Biomphalaria* snail hosts [34,95] on the rate or extent of *S*. *mansoni* development, it is not surprising that field-derived representatives will differ with respect to extent of parasite development and transcriptional activity. Here it should be noted that read counts may not always be fully indicative of *S*. *mansoni* biomass in snails as the transcriptional activity of the parasite may vary temporally, both daily [96] and at longer time scales [97], and in response to other stimuli, as noted in the following section regarding symbionts.

### Recovered symbiont sequences

Whole snail transcriptome sequencing gave us the opportunity to identify sequences of non-mollusc and non-schistosome origin, including viruses, bacteria and eukaryotes. These sequences provide evidence of symbionts that are found in or on *B*. *pfeifferi* and/or *S*. *mansoni*. Some of the symbionts identified are surely worthy of further future investigation and may offer potential in application of novel and as yet unforeseen control efforts.

With respect to viruses, in general the array of viruses found in invertebrates has recently been shown to be much more diverse than previously known, including in molluscs [98]. Of the nearly 1,300 of our assembled CDS identified provisionally as viruses, most have homology to Beihai paphia shell viruses, picorna-like viruses, and crawfish viruses. In terms of read abundance, the five most abundant viral CDS we found in *B*. *pfeifferi* had the most similarity to the Wenzhou picorna-like virus 33 from the channeled apple snail *Pomacea canaliculata*, Sanxia picorna-like virus 4 from a freshwater atyid shrimp, Beihai picorna-like virus 47 from a sesarmid crab, bivalve RNA virus G2 a picorna virus from the gills of a bivalve [99], and Beihai hypo-like virus 1 from a razor shell [98]. Picorna viruses have recently been described in both *B*. *glabrata* from South America and *B*. *pfeifferi* from Oman [100]. Three novel RNA viruses were reported in the *B*. *glabrata* genome, the first with similarities to an iflavirus, the second with similarities to a Nora virus or Picornavirales, and the third with similarities to several viruses [37]. Further study is required to confidently designate any of the putative viral sequences recovered as actual infectious entities of snails, or possibly of schistosomes or other digeneans. They might infect other potential hosts like rotifers or diatoms among the symbionts living in *B*. *pfeifferi*.

The recovery of a few sequences of the digenean-inhabiting *Neorickettsia* from two infected snails with relatively high percentages of *S*. *mansoni* reads (3d-R3 and shedding-R1) is suggestive of an association. *Neorickettsia* has been found from non-human schistosomes [101] but further study is needed to document the presence of *Neorickettsia* in human-infecting schistosomes. For example, the *Neorickettsia* might be associated with metacercariae of other digeneans that are commonly found encysted in *B*. *pfeifferi* from natural habitats.

With respect to eukaryotes, CDS representing the following groups were recovered: 1) *Chaetogaster* annelids which mostly colonize the external soft surfaces of freshwater snails and are known to ingest digenean miracidia and cercariae [102–105]; 2) *Trichodina* ciliates known to live on the soft surfaces of snails but with poorly characterized influence on their snail hosts [106]; 3) *Capsaspora owczarzaki*, a Filasterean amoeba-like symbiont known from *Biomphalaria glabrata* [107,108]; 4) Microsporidians, not surprising for *B*. *pfeifferi* considering microsporidians are known from both *Biomphalaria* and *Bulinus* [109]; 5) Perkinsea, an alveolate group of considerable commercial significance in marine bivalves, but with at least two reports suggesting their presence in freshwater habitats as well [110,111]; 6) Rotifers (possibly attached to the shell or ingested) and diatoms (probably ingested) were frequently recovered as well; 7) Four tardigrade CDS were recovered, two from the uninfected control 454-sequenced snail similar to *Richtersius coronifer* and two from the Illumina *de novo* assembly similar to *Ramazzottius varieornatus*. Control-R1 had read counts >10 for the two *R*. *coronifer* CDS and 1d-R3 had read counts >10 for a *R*. *varieornatus* CDS. It is not unprecedented to find tardigrades associated with snails. Fox and García-Moll [112] identified the tardigrade *Echiniscus molluscorum* in the feces of land snails from Puerto Rico. Although the tardigrade may have been ingested along with food, the authors did not rule out the possibility that *E*. *molluscorum* may be a symbiont of the snail.

It was not surprising that two of our snails yielded several reads mapping to sequences from other digeneans. The first, control-R3, returned sequences consistent with *Ribeiroia*, representatives of which occur in East Africa and are known to infect *Biomphalaria* there [91]. It seems most likely this snail had an infection with *Ribeiroia* sporocysts and/or rediae, though the extent of this infection must have been minimal as the transcriptomics response of this snail was not unusual compared to the other control snails. It may also have been infected with *Ribeiroia* metacercariae which are most familiarly known to infect amphibians or fish [113,114], but have been recovered and sequence-verified in specimens of *Biomphalaria spp*. from Kenya (MR Laidemitt, personal communication, April 2017). The other snail, 3d-R2, yielded confirmed amphistome sequences, probably from the commonly recovered species *Calicophoron sukari* [91], so it may have harbored developing larvae of both *S*. *mansoni* and an amphistome, reflective of real-life circumstances in the habitat of origin where this amphistome species is the most common digenean to infect *B*. *pfeifferi* [92]. This co-infection may help to explain the relatively low numbers of *S*. *mansoni* reads recovered from this snail relative to 3d-R1 and 3d-R3. It has also been noted that *B*. *pfeifferi* ingests amphistome metacercariae (A Gleichsner, personal communication, June 2017) which are abundant on the submerged vegetation in the habitat from which the snail was collected, so this may be an alternative explanation for the presence of amphistome reads in 3d-R2. The peculiar nature of infection in this snail further justifies our rationale for including it in the separate analyses (3v1) described in the results.

### Some overall highlights of the response of infection

At 1d, snails showed proportionately more down-regulated CDS, possibly reflective of a strong parasite-induced immunomodulatory effect during the establishment phase of infection [54]. For the two additional time points examined, the majority of features in *B*. *pfeifferi* were up-regulated (Fig 8; S3 File). This pattern differed from a previous microarray-based expression studies for susceptible *B*. *glabrata* for which a predominant trend of down-regulation was noted from 2–32 days post-exposure to *S*. *mansoni* [47]. The more comprehensive transcriptional picture resulting from next-gen sequencing provides a different overview of responses following infection with *S*. *mansoni* (see also [54]).

Many host CDS responded uniformly across individual snails regardless of the number of *S*. *mansoni* reads recovered. However, at 1d and 3d, snails with fewer *S*. *mansoni* reads had higher proportions of up-regulated features than did snails with higher numbers of *S*. *mansoni* reads. Furthermore, for both 3d and shedding snails, snails with low *S*. *mansoni* read counts had smaller proportions of down-regulated features. These patterns are suggestive that up-regulated host responses might limit *S*. *mansoni* gene expression and that snails with less parasite gene expression may be less vulnerable to gene down-regulation, but care in interpretation is required as alternative explanations may exist. For example, as noted above, replicate 3d-R2 also contained an amphistome infection. Negative interactions among the two digeneans which are known to occur from experimental studies (MR Laidemitt, personal communication, April 2017) may account for the limited number of *S*. *mansoni* reads.

At 1d, up-regulated responses, as exemplified by CDS for phospholipases, endoglucanases, and several proteases and protease inhibitors, were usually less pronounced than at 3d, suggesting it takes a few days to mobilize responses. Notable at 1d were down-regulation of CDS that might lower hemoglobin levels, and influence feeding behavior and heart beat rate. Infected snails exhibited complex mixed responses with respect to mucins, multidrug resistance proteins, glutathione-S-transferases and cytochrome P450 family members. Cytochrome P450s are part of the stress response shown by *B*. *glabrata* snails following exposure to molluscicides [49] and to biotic stressors [48]. For heat shock proteins, *B*. *glabrata* snails elaborated more complex up-regulated responses following exposure to molluscicides [49] than *B*. *pfeifferi* did following exposure to *S*. *mansoni*. Complex patterns in stress response gene families were also noted for 3d and shedding snails. It is noteworthy that exposure to *S*. *mansoni*, a specific extrinsic biotic stressor, also provokes components of a generalized stress response in *B*. *pfeifferi* and *B*. *glabrata* [115,116].

Snails with 3 day infections had the highest number of up-regulated CDS. Some of the features down-regulated at 1d were again down at 3d. Additionally, one CDS (aryl hydrocarbon receptor) associated with controlling circadian rhythm [117] was down-regulated. Daily feeding patterns of infected snails [119–121] or patterns of release of cercariae [96] could potentially be influenced by this CDS. Several gene families also showed complex patterns of responses at 3d. Among them were amine oxidases which, as noted by Zhang *et al*. [48], are involved in oxidation of amine-containing compounds including neurotransmitters, histamines and polyamines [122].

The overall responses of shedding snails were surprising in not being more dramatically altered relative to controls than they were. This is because snails with more advanced schistosome infections (28+ day infections) experience several noteworthy physiological changes, including altered feeding behavior, decreased locomotory activity, increased heartbeat rate [118–121,123] and castration (see section below). From our shedding snails, we noted up-regulated levels of FMRF-amide receptor and small cardioactive peptides that influence heart beat rate. Shedding snails also uniquely showed up-regulated levels of CDS involved in collagen synthesis or epithelial cell and blood vessel formation, processes involved in wound healing [49,123,124], of relevance to a snail experiencing the tissue damage associated with cercarial emergence. Other up-regulated features are indicative of stress. Modestly up-regulated levels of reverse transcriptase are of interest because of previous reports of enhanced RT activity in susceptible *B*. *glabrata* exposed to *S*. *mansoni* [115].

Down-regulated levels of features potentially helping to explain reduced growth rates [125,126], reduced motility [119,120,127,128] or depleted levels of hemoglobin [129] observed in shedding snails were noted (S3 File). Other down-regulated features of interest were noted including tyrosinase, which is involved in melanin synthesis (see also discussion of reproduction).

### Consequences of infection on host reproduction

Snails infected with the proliferating larval stages of digenetic trematodes, including *B*. *pfeifferi* infected with *S*. *mansoni*, suffer parasitic castration, marked by a sharp or complete reduction in production of eggs [121,125,130]. In *B*. *pfeifferi*, egg-laying begins to decline 7–10 days following exposure to *S*. *mansoni* and is complete in most snails by 14 days. The time course and extent of castration are influenced by the age of the snail at the time of exposure and by the dose of miracidia received [130,131]. In some cases, a slight increase in egg production compared to unexposed controls can be seen in the pre-shedding period, but this is followed by castration [125,130,131].

Studies of the reproductive physiology of freshwater gastropods have identified a number of peptides and non-peptide mediators (including biogenic monoamines) involved in neuro-endocrine control of reproduction [132,133]. We found evidence for the presence and expression of homologs of over 50 of these neuropeptides in *B*. *pfeifferi* (S4 File; Fig 9) and several additional neuropeptide precursors. It has also been noted that in *B*. *glabrata* castrated by *S*. *mansoni*, repeated exposure to serotonin enabled snails to resume egg-laying [134]. Furthermore, dopamine is present in reduced levels in infected snails, and administration of this catecholamine stimulated the release of secretory proteins from albumen gland cultures of *B*. *glabrata* [135] and the related snail *Helisoma duryi* [136].

Although infections of 1 or 3 days duration are too young to manifest castrating effects, up-regulation of some features with possible inhibitory effect on reproduction were noted at these times. Several features were also down-regulated at 1 day, including ovipostatin 2, a type 1 serotonin receptor (relevant because of serotonin’s ability to stimulate egg-laying), and schistosomin. Schistosomin has been implicated in *Lymnaea stagnalis* in inhibiting hormones involved in stimulating egg-laying or the albumen gland [137]. A role for schistosomin in reproduction or trematode-mediated castration was not found in *B*. *glabrata* infected with *S*. *mansoni* [138] and we saw no change in its expression in *B*. *pfeifferi*. Kynurenine 3-monooxygenase-like transcripts were up-regulated in all snails with 3 day infections. By degrading tryptophan, this enzyme may limit concentrations of serotonin.

It was of interest to learn if the water-borne pheromones (temptin, enticin, seduction, and attractin) that favor aggregation in *Aplysia* [139] were expressed in *B*. *pfeifferi*, especially given its preference for self-fertilization. We found evidence only for the expression of temptin, which was up-regulated at 3d, but otherwise was not differentially expressed. Likewise, only temptin was isolated in proteins released from *B*. *glabrata* [37] and egg-mass proteins [140]. It has been shown to be an attractant for *B*. *glabrata* [141].

Our results with shedding snails are most pertinent with respect to parasitic castration. Several reproduction-related neuropeptides, including caudal dorsal cell hormone, and neuropeptides associated with production of egg and egg mass fluids such as snail yolk ferritin (vitellogenin), galactogen synthesis, lipopolysaccharide binding protein/bacterial permeability-increasing proteins (LBP/BPI) or aplysianin/achacin-like protein [140] were not strongly down-regulated as a consequence of infection. Some of the most obvious changes we noted were up-regulated levels of transcripts encoding dopamine beta hydroxylase and especially dopamine beta-hydroxylase–like monooxygenase protein 1, both of which convert dopamine to noradrenaline so their enhanced expression may help to explain the declining levels of dopamine noted in *S*. *mansoni*-infected snails [134]. This may in turn help to explain diminished egg production given dopamine’s effect on release of albumen gland proteins. Tyrosinase-1, involved in production of melanin, is down-regulated in shedding snails and this may have the effect of preserving dopamine levels in these snails. At both earlier sampling points, tyrosinase-1 is strongly up-regulated especially in snails with abundant *S*. *mansoni* reads, and thus may mark an early phase in initiation of castration by diverting tyrosine to production of melanin as opposed to dopamine. Transcription of enzymes involved in dopamine metabolism are strongly affected in *S*. *mansoni*-infected snails. Tyrosinase-1 is also discussed in the next section regarding its potential involvement in defense responses.

There are numerous ovipostatins produced in *Biomphalaria* (we found 6 different versions in *B*. *pfeifferi*), with ovipostatin 5 being the most prominent responder in shedding snails. In *L*. *stagnalis*, ovipostatin is passed in seminal fluid from one individual to another during mating and inhibits oviposition in the recipient [132]. Although *B*. *pfeifferi* is predominantly a self-fertilizer [20], ovipostatin 5 could potentially down-regulate oviposition in ways not reliant on copulation. Neuropeptide Y inhibits egg-laying in *L*. *stagnalis* [142] and though we did not observe up-regulation of this neuropeptide, up-regulated transcripts for neuropeptide Y receptor type 5-like protein in our shedding snails is consistent with a possible enhanced inhibitory effect on reproduction of neuropeptide Y. Strong up-regulation of transcripts for yolk ferritin-like and snail yolk ferritin molecules (vitellogenins) in shedding snails was also observed and is somewhat paradoxical but may suggest they are diverted to the parasite for metabolism since it is known that schistosomes require iron stores for development [143]. Notably, the extent of up-regulation for yolk ferritin-like and snail yolk ferritin, ovipostatin 5, neuropeptide Y receptor type 5-like, and dopamine beta-hydroxylase-like, was the least in the shedding snail expressing the lowest number of normalized *S*. *mansoni* reads.

Wang *et al*. [133] recently used proteomics methods (liquid chromatography tandem mass spectrometry) to examine and identify neuropeptides in central nervous system (CNS) ganglia dissected from *B*. *glabrata*, either from control snails or snails at 12 days post infection with *S*. *mansoni*. They noted many reproductive neuropeptides, such as egg laying hormone 2, at significantly reduced levels at 12d compared to controls. They also reported an increase in some neuropeptides including FMRFamide, luqin, NKY, and sCAP in infected snail CNS. Based on predicted protein interaction networks, Wang *et al*. [133] suggested that snail-produced leucine aminopeptidase 2 (LAP2) interacts with several *S*. *mansoni* miracidia peptides so may be a key player in regulating parasite-induced changes in host physiology. A homolog to the *B*. *glabrata* LAP2 was present in our transcriptome but was not differentially expressed in any sample. When comparing our results to those of Wang *et al*. [133], it should be noted that our approach was transcriptome-centered, examined different time points post-infection, and was based on whole body extractions of *B*. *pfeifferi*, rather than *B*. *glabrata*. Our methods may bias against detection of changes in expression of potentially rare neuropeptide transcripts, but cast a wider net for potential downstream effects of castration, so provides a valuable complementary view to the approach taken by Wang *et al*. [133].

### Immune response of *B*. *pfeifferi* infected with *S*. *mansoni*

At 1d (S4 File; Fig 10), several immune-relevant CDS were up-regulated in all three snails including dermatopontins (frequently noted in *B*. *glabrata* studies), ficolins [48], and chitinase attacking enzymes [42,48]. For the two snails with the highest proportions of *S*. *mansoni* reads, up-regulated responses were observed for a number of additional immune features. Cu,Zn SOD is of particular interest because previous work has implicated high expression of certain alleles of Cu,Zn SOD with resistance to *S*. *mansoni* in the 13-16-R1 strain of *B*. *glabrata*, because of Cu,Zn SOD’s involvement in converting superoxide anion to schistosomicidal hydrogen peroxide [144–146]. Our study is in agreement with Hanington *et al*. [47] who noted up-regulated levels of Cu,Zn superoxide dismutase (SOD) at early time points following exposure of susceptible M line *B*. *glabrata* to either *S*. *mansoni* or *E*. *paraensei*.

Hanington *et al*. [47] also found both FREP2 and FREP4 to be consistently up-regulated following exposure of M line *B*. *glabrata* to *S*. *mansoni* or *E*. *paraensei*, so much so either might be considered as markers of infection. Although a FREP2 homolog was consistently up-regulated following exposure of *B*. *pfeifferi* to infection, a FREP4 homolog was not expressed in *B*. *pfeifferi* at any of the time points we examined.

In contrast, among CDS more up-regulated in the 1 day infected snail with a low proportion of *S*. *mansoni* reads were macrophage expressed gene-1, known to be up-regulated in both abalone following bacterial infection [147] and from resistant and non-susceptible strains of *B*. *glabrata* in early exposure to *S*. *mansoni* [148]. Hemocytin was also up-regulated in the 1d snail with low proportion of *S*. *mansoni* reads. Hemocytin, a homolog for an insect humoral lectin with a role in hemocyte nodule formation [149], was consistently up-regulated in all *S*. *mansoni*-infected snails at all three time points, especially at 3d when it was increased over 8-fold in expression. For both 1 and 3d, hemocytin expression was highest in those snails with fewer *S*. *mansoni* reads. FREP3, previously implicated in resistance to *S*. *mansoni* in *B*. *glabrata* [52], was minimally responsive in this compatible *B*. *pfeifferi* system. It was modestly up-regulated only at 1d, in the snail with fewest *S*. *mansoni* reads.

Down-regulated immune features at 1d were relatively few but prominent among them were FREP12 and its precursors, toll-like receptor 8 and cytidine deaminase. FREP12 down-regulation has also been noted upon exposure of *B*. *glabrata* amebocyte-producing organs to fucoidan, a fucosyl-rich PAMP chosen to mimic the surface of *S*. *mansoni* sporocysts [48], and in *B*. *glabrata* exposed to *S*. *mansoni* [47]. A strain of *B*. *glabrata* resistant to *S*. *mansoni* exhibits higher levels of a TLR on its immune cells, and exposure to *S*. *mansoni* significantly enhances their expression, whereas compatible snails show little response following exposure to infection [31]. Our *B*. *pfeifferi* showed no conspicuously up-regulated TLR genes at 1d, and we found no *B*. *pfeifferi* TLR with strong homology to the TLR reported by Pila *et al*. [31], but the relatively strong down-regulation of TLR 8 in this model of compatibility is noteworthy. Although the immune role of cytidine deaminase is not clear, Bouchut *et al*. [150] associated higher levels of its expression with enhanced resistance to echinostome infections and Ittiprasert *et al*. [148] observed up-regulation of cytidine deaminase in resistant and non-susceptible *B*. *glabrata* in early exposure to *S*. *mansoni*. Down-regulation of cytidine deaminase might therefore be associated with lower responsiveness to *S*. *mansoni* infections, particularly early in infection (down-regulation also noted at 3d, but not in shedding snails).

The responses of putative immune factors were most extensive in snails at 3d and this is not surprising as this is a critical stage in the early establishment of the parasite. Several CDS mentioned with respect to the 1d response were again noted at 3d. Snails with more *S*. *mansoni* reads had high levels of several transcripts including for aplysianin-like proteins and FREP 5. Aplysianin, first described from *Aplysia*, is an L-amino oxidase that has tumoricidal and bactericidal effects [151], and a distinct aplysianin-like protein exists in egg mass fluids of *B*. *glabrata* [140]. Aplysianin-like transcripts were more abundant in echinostome-resistant than susceptible strains of *B*. *glabrata* [150]. FREP 5 was shown to be down-regulated in microarray studies of *B*. *glabrata* in response to successfully developing *S*. *mansoni* or *Echinostoma paraensei* [47].

The snail with relatively few *S*. *mansoni* reads at 3d revealed a different group of up-regulated transcripts, with hemocytin again being prominent. Also notable were distinctive CDS potentially involved in hemocyte aggregation [152], FREP 7, peptidoglycan-recognition proteins SC2-like (PGRPs), and TLR 13. PGRPs are well-known anti-bacterial factors and were found to be up-regulated following exposure of *B*. *glabrata* to LPS [153] and to bacteria [53]. Down-regulated features for snails with 3d infections again included cytidine deaminase, FREP12 precursors, and TLR 4 and 8 among others.

Laccases and tyrosinases are two groups of phenoloxidases observed to be responsive in early *S*. *mansoni* infection within *B*. *pfeifferi* (Table 6; S4 File). Tyrosinase has been isolated from *B*. *glabrata* egg masses with a presumptive immunoprotective effect for offspring [140,154]. As mentioned earlier with details of its reproductive consequences, tyrosinase-1 was up-regulated at 1d and 3d. Tyrosinase-1 was down-regulated in the shedding replicate with the least *S*. *mansoni* reads and tyrosinase-3 was down-regulated in the two replicates with the most *S*. *mansoni* reads. Another type of phenoloxidase, laccase, was shown to have decreased activity in *B*. *glabrata* hemolymph beginning at 7 weeks post-infection with *S*. *mansoni* [155]. We found laccase-15-like was up-regulated in all three comparisons (3v3, 3v2, 3v1) at both 1d and 3d. Laccase-1-like was up-regulated in all three comparisons at 1d and laccase-2-like was up-regulated in all comparisons at 3d. Laccases were not significantly DE in shedding snails. In *B*. *pfeifferi*, the up-regulation of two phenoloxidases, tyrosinase and laccase, at 1d and 3d suggests an increase in the synthesis of early-stage reactions in the melanin pathway, however, further work is needed to determine if melanization is involved in schistosome killing, especially in the *B*. *pfeifferi* model characterized by its compatibility.

It is worth noting that members GTPase IMAP family (GIMAP) were found to be up-regulated in 1d and 3d (mostly up-regulated in 3d). The possible role of GIMAPs in immunity has not been realized in protostomes until it was shown that several GIMAPs were up-regulated in the amebocyte organ of *B*. *glabrata* following exposure to extrinsic stimuli [48]. This finding was reconfirmed by later work, which demonstrated that GIMAPs not only play a role in immunity, but are highly diverse in the eastern oyster *Crassostrea virginica* where they were down-regulated following exposure to bacterial infection. GIMAPs may promote hemocyte survival by inhibiting apoptosis [156].

Immune-related responses for shedding snails were surprising for being mostly up-regulated (Fig 10), with only a few features being modestly down-regulated, among them galectin-6. Galectins recognize carbohydrates associated with schistosome surfaces and are implicated as pattern recognition receptors for other pathogens as well [157]. Dihydropyrimidinase and cytidine deaminase, also down-regulated, are additional CDS potentially affecting pyrimidine levels in infected snails. Interestingly, in contrast to 1d and 3d responses, shedding snails did not show up-regulated Cu,Zn SOD levels.

Among those up-regulated features, shedding snails with high levels of *S*. *mansoni* reads had distinctly higher responses for aplysianin-A-like, beta-1,3-glucan binding protein-like, and FREPS 2, 7 and 14. By contrast, the snail with a low percentage of *S*. *mansoni* reads expressed higher levels of macrophage-expressed gene, chitinase-3-like-protein, a distinct CDS with a leucine rich repeat and immunoglobulin domain, and TLR 3.

Features highlighted in recent genetic linkage studies [32,50,51] including components of the “Guadeloupe Resistance Complex” were sought among *B*. *pfeifferi* transcripts. Most did not show strong patterns of up- or down regulation in this compatible species following exposure to *S*. *mansoni*, but zinc metalloproteinase/disintegrin-like was down-regulated at 1d and zinc metalloproteinase nas-13- and -14-like showed some up-regulation in shedding snails. Probable serine carboxypeptidases (versions 1–5) revealed a mixed pattern of expression at 3d, but were mostly up-regulated, whereas probable serine carboxypeptidase CPVL was down-regulated in shedding snails. Granulin, a growth factor that drives the proliferation of immune cells was up-regulated at both 1d and 3d [30,33].

### Genes showing either extraordinary up- or down-regulation following exposure to *S*. *mansoni* infection

Unlike *B*. *glabrata* for which isolates or inbred lines are known that are resistant to *S*. *mansoni*, *B*. *pfeifferi* is a species typically discussed in the context of its high compatibility with many *S*. *mansoni* isolates. Although particular lineages of *B*. *pfeifferi* may certainly come to light that exhibit strong incompatibility, key factors that dictate compatibility might best be sought not among the putative immune factors that characterize the *B*. *glabrata* response, but among those genes that exhibit the strongest transcriptional responses, up or down, to *S*. *mansoni* exposure (Table 5; S3 File). Strongly up-regulated snail genes may be responsible for encoding proteins essential to *S*. *mansoni* development, and those strongly down-regulated may represent parasite-manipulated factors that if left un-manipulated would otherwise discourage parasite development. Certainly such a role has been proposed for schistosomes in altering expression of genes in compatible snails to their advantage [158–160].

Although many B. pfeifferi CDS that were highly altered in their expression are unknowns and thus represent intriguing subjects for future research, some did have homologs in the database and could also represent outstanding future targets for manipulation to discourage *S*. *mansoni* development. For example, we note the up-regulation of the protease inhibitor papilin-like and galactocerebrosidase-like. Galactocerebrosidase is an enzyme that removes galactose from galactocerebrocide (a ceramide sphingolipid with a galactose residue) to form a ceramide, an important lipid signaling molecule that has been reported in *Crassostrea gigas* [161]. A transcript coding for deleted in malignant brain tumor 1 protein-like (DMBT1) was also highly up-regulated in all shedding snails. DMBT1 is a pattern recognition receptor in mammals that belongs to a group of secreted scavenger receptors involved in pathogen binding [162]. However, its role in invertebrate systems needs to be established [159].

In conclusion, provided here is a de novo assembled transcriptional database based on over half a billion paired-end reads for an understudied schistosome vector, *B*. *pfeifferi*, one that is probably responsible for transmission of more *S*. *mansoni* to people than any other *Biomphalaria* species. We have deliberately chosen to emphasize the study of field-derived *B*. *pfeifferi* and *S*. *mansoni* to provide a more realistic view of the context in which they live, and how they interact in the wild, including with third party symbionts. Our approach has revealed that the extent of *S*. *mansoni* transcriptional activity varies among snails and this is reflected in different transcriptional responses of the snails, suggestive of diverse trajectories in what is typically a highly compatible host-parasite model. We have highlighted several snail features warranting further study with respect to their roles in potentially supporting or enabling parasite development, that might limit the extent of development, and that might play a role in the diminished egg production typically shown by snails with shedding *S*. *mansoni* infections. Another generation of research exploiting the power of techniques like CRISPR-Cas, when it becomes available for snails, will enable further dissection of the functional role of these candidate molecules. A further challenge will then be to determine how the responses of compatible snails, or perhaps of the schistosome parasites within, can be exploited, ideally to prevent or suppress in a highly specific manner the development of schistosome parasites in snails.

## Supporting information

S1 Table qPCR primers used to validate differential expression trends. (DOCX)

S2 Table *Biomphalaria pfeifferi* CDS identified as a symbiont of interest during annotation efforts and verified by MEGABLAST x NCBI nt database. (DOCX)

S3 Table *Biomphalaria pfeifferi* CDS with a BLASTn hit against publicly available genomes and CDS from molluscan symbionts of interest. (DOCX)

S1 Fig Nucleotide sequence length distribution of 194,344 assembled *B*. *pfeifferi* CDS from EvidentialGene. (TIF)

S2 Fig The top 20 most abundant Gene Ontology assignments for *B*. *pfeifferi* CDS in each molecular function, cellular component category, and biological process. (TIF)

S3 Fig All represented KEGG classes, organized by category and abundance, in the *B*. *pfeifferi* transcriptome. (TIF)

S4 Fig BLASTp distribution of *B*. *pfeifferi* ORFs that have homologs to *B*. *glabrata* predicted polypeptides. (TIF)

S5 Fig Relationships of selected molluscs with genomes and/or transcriptomes sequenced. Percentages next to organisms show the percent of proteins present in our *B*. *pfeifferi* transcriptome predicted by TransDecoder.
(TIF)

S1 File Annotations for *B*. *pfeifferi* transcriptome. (XLSX)

S2 File Read counts for symbionts of interest. (XLSX)

S3 File Overall differential expression results for *B*. *pfeifferi*. (XLSX)

S4 File General, reproduction or immune system features that were most differentially expressed. (XLSX)
