# Supplementary material for: Transcriptomic responses of Biomphalaria pfeifferi to Schistosoma mansoni: Investigation of a neglected African snail that supports more S. mansoni transmission than any other snail species
Source: PLoS Negl Trop Dis. 2017 Oct 18;11(10):e0005984. doi: 10.1371/journal.pntd.0005984 (PMC5685644; doi:10.1371/journal.pntd.0005984)
Supplement: S1 Table — (DOCX) [file pntd.0005984.s001.docx]

|  | **TARGET GENE** | **TARGET *B. pfeifferi* CDS** | **NAME** | **TEMP** | **E** | **R^2** | **SEQUENCE (5'-3')** |
| --- | --- | --- | --- | --- | --- | --- | --- |
| **Reference** | 40S ribosomal protein S2 | evgTRINITY_DN94486_c0_g1_i1 | S2_99-F | 58°C | 99.2% | 0.999 | GCCATCCGAGGAGCTATC |
|  |  |  | S2_99-R |  |  |  | GCAGGGAACAGTGTGAGG |
|  | elongation factor 1-alpha | evgTRINITY_DN94461_c0_g1_i1 | EF1_124-F | 58°C | 104.7% | 0.997 | CCAGTCGGCAGAGTTGAG |
|  |  |  | EF1_124-F |  |  |  | GGGTGGCTTCCTGAAGAG |
| **Experimental** | basic salivary proline-rich protein 1-like isoform X2 | evgTRINITY_DN93030_c30_g1_i1 | Sal_148-F | 56°C | 90.0% | 0.989 | TGGCTGTCTTCCTTACAAGC |
|  |  |  | Sal_148-R |  |  |  | TTGCTCGAGCTTTTGGTC |
|  | complement C1q-like protein 4, partial | evgTRINITY_GG_443_c0_g2_i1 | C1q4_151-F | 58°C | 101.8% | 0.995 | GCTCTACCTGTTCCAGTTCC |
|  |  |  | C1q4_151-R |  |  |  | AGCCAGCTTCAGGATTACAG |
|  | cell wall protein DAN4-like | evgTRINITY_DN88408_c0_g1_i1 | DAN4_156-F | 56°C | 101.4% | 0.998 | GATGTTGTCCAGATGCAGTG |
|  |  |  | DAN4_156-R |  |  |  | GAGTCGATGTTTCCTCAACC |
|  | angiotensin-converting enzyme-like, partial | evglcl\|G0WVJSS02GCJC6 | Ang_190-F | 54°C | 99.5% | 0.998 | TGCTTGCTGGAGTCAGTAGTC |
|  |  |  | Ang_190-R |  |  |  | AACTGATCCCACTTGAGCTG |
