# Supplementary material for: Transcriptomic responses of Biomphalaria pfeifferi to Schistosoma mansoni: Investigation of a neglected African snail that supports more S. mansoni transmission than any other snail species
Source: PLoS Negl Trop Dis. 2017 Oct 18;11(10):e0005984. doi: 10.1371/journal.pntd.0005984 (PMC5685644; doi:10.1371/journal.pntd.0005984)
Supplement: S2 Table — (DOCX) [file pntd.0005984.s002.docx]

| **MEGABLAST IDENTIFICATION** | **GI** | ***B. pfeifferi* CDS** | **% ID** | **% Query Coverage** |
| --- | --- | --- | --- | --- |
| *Chaetogaster diastrophus* 28S rRNA | GQ355444.1 | evglcl\|HJ4YRIA02J27K8 | 93% | 100% |
| *Chaetogaster diastrophus* 28S rRNA | GQ355444.1 | evglcl\|HJ4YRIA02GT5FU | 91% | 100% |
| *Chaetogaster diastrophus* 28S rRNA | GQ355444.1 | evglcl\|HJ4YRIA01CKT7N | 91% | 100% |
| *Chaetogaster diastrophus* 28S rRNA | GQ355444.1 | evglcl\|HJ4YRIA02IR0J2 | 91% | 99% |
| *Chaetogaster diastrophus* 28S rRNA | GQ355444.1 | evglcl\|HJ4YRIA02FUU8R | 90% | 100% |
| *Chaetogaster diastrophus* 28S rRNA | GQ355444.1 | evglcl\|HJ4YRIA01ALBT8 | 92% | 93% |
| *Trichodina* *sp.*18S rRNA | KP295473.1 | evglcl\|HJ4YRIA02F8S6H | 100% | 93% |
| *Trichodina* *sp.* 18S rRNA | KP295473.1 | evgTRINITY_GG_454Inf_840_c12_g1_i1 | 99% | 100% |
| *Trichodina* *sp.* 18S rRNA | AY363960.1 | evgTRINITY_DN54951_c0_g1_i1 | 99% | 100% |
| *Paenibacillus sp.* | CP00928.1 | evglcl\|HJ4YRIA02HHYCK | 90% | 95% |
| *Paenibacillus* sp. | CP018620.1 | evglcl\|HJ4YRIA01AG2P6 | 82% | 99% |
| *Paenibacillus sp.* | CP018620.1 | evglcl\|HJ4YRIA01AHR45 | 81% | 99% |
| *Neorickettsia sp.* 3225 16S rRNA | KX818103.1 | evglcl\|HJ4YRIA02JQFT3 | 99% | 100% |
| *Microsporidium sp.*  BIOB 16S rRNA, ITS1 | AJ871391.1 | evglcl\|HJ4YRIA02GDN25 | 100% | 100% |
| *Microsporidium sp.*  BIOPC 16S rRNA | AJ871390.1 | evglcl\|HJ4YRIA02FYGW4 | 99% | 100% |
| *Microsporidium sp.*  BIOPC 16S rRNA | AJ871390.1 | evglcl\|HJ4YRIA02H8J2W | 99% | 100% |
| *Microsporidium sp.*  BUL 16S rRNA | AJ871392.2 | evgTRINITY_GG_454Inf_715_c1_g1_i1 | 99% | 99% |
| *Microsporidium* *sp.* BUL 16S rRNA | AJ871392.2 | evglcl\|HJ4YRIA02ID8PW | 99% | 100% |
| *Microsporidium sp.*  BUL 16S rRNA | AJ871392.2 | evgTRINITY_GG_454Inf_415_c15_g1_i1 | 100% | 100% |
| *Microsporidium sp.*  BUL 16S rRNA | AJ871392.2 | evglcl\|HJ4YRIA01B442V | 99% | 100% |
| *Microsporidium sp.*  BUL 16S rRNA | AJ871392.2 | evglcl\|HJ4YRIA01ATBOD | 99% | 100% |
| *Microsporidium sp.*  BUL 16S rRNA | AJ871392.2 | evglcl\|HJ4YRIA02GPVDH | 99% | 100% |
| *Microsporidium sp.*  HEM-2006MCD 16S rRNA | AM411637.1 | evglcl\|HJ4YRIA02FZ7N4 | 97% | 99% |
| *Microsporidium sp.*  HEM-2006MCD 16S rRNA | AM411637.1 | evglcl\|HJ4YRIA01B7MY3 | 98% | 100% |
| *Microsporidium sp.*  HEM-2006MCD 16S rRNA | AM411637.1 | evglcl\|HJ4YRIA01C1BLC | 99% | 100% |
