# Supplementary material for: Transcriptomic responses of Biomphalaria pfeifferi to Schistosoma mansoni: Investigation of a neglected African snail that supports more S. mansoni transmission than any other snail species
Source: PLoS Negl Trop Dis. 2017 Oct 18;11(10):e0005984. doi: 10.1371/journal.pntd.0005984 (PMC5685644; doi:10.1371/journal.pntd.0005984)
Supplement: S3 Table — (DOCX) [file pntd.0005984.s003.docx]

| **Species of Interest** | | **Data File** | **# *B. pfeifferi* CDS** |
| --- | --- | --- | --- |
|  | *Capsaspora owczarzaki* | Capsaspora_owczarzaki_atcc_30864.C_owczarzaki_V2.cds.all [85] | 103 |
|  | *Capsaspora owczarzaki* | Capsaspora_owczarzaki_atcc_30864.C_owczarzaki_V2.dna.toplevel [85] | 216 |
|  | *Perkinsus marinus* | Perkinsus_marinus_atcc_50983.JCVI_PMG_1_0.cds.all | 121 |
|  | *Perkinsus marinus* | Perkinsus marinus ATCC 50983 genome | 257 |
| **Microsporidians** [86-89] | *Anncalliia algerae* | anncaliia_algerae_pra109_4 | 1 |
|  | *Anncalliia algerae* | anncaliia_algerae_pra339_2 | 1 |
|  | *Edhazardia aedis* | edhazardia_aedis_usnm_41457_1 | 13 |
|  | *Encephalitozoon cuniculi* | encephalitozoon_cuniculi_eci | 26 |
|  | *Encephalitozoon cuniculi* | encephalitozoon_cuniculi_ecii | 36 |
|  | *Encephalitozoon cuniculi* | encephalitozoon_cuniculi_eciii | 6 |
|  | *Encephalitozoon cuniculi* | encephalitozoon_cuniculi_gb-m1 | 3 |
|  | *E. intestinalis* | encephalitozoon_intestinalis_atcc_50506 | 1 |
|  | *Nematocida sp* | nematocida_sp1_ertm2_1 | 4 |
|  | *Nematocida sp* | nematocida_sp1_ertm6_2 | 4 |
|  | *Vavraia culicis* | vavraia_culicis_floridensis_1 | 12 |
|  |  |  |  |
