# Supplementary figures and images for: Transcriptomic responses of Biomphalaria pfeifferi to Schistosoma mansoni: Investigation of a neglected African snail that supports more S. mansoni transmission than any other snail species
Source: PLoS Negl Trop Dis. 2017 Oct 18;11(10):e0005984. doi: 10.1371/journal.pntd.0005984 (PMC5685644; doi:10.1371/journal.pntd.0005984)

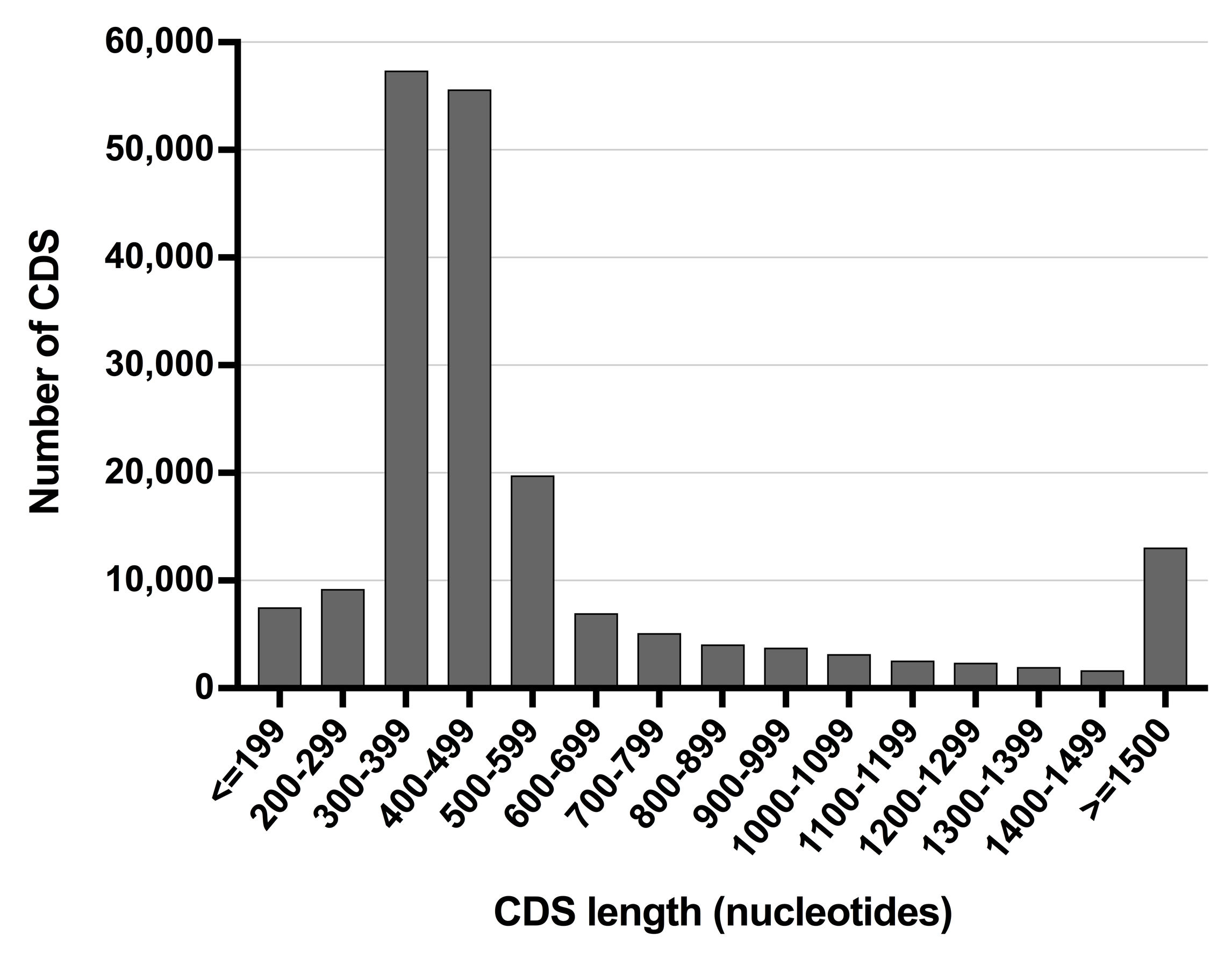

Supplement: S1 Fig — (TIF) [file pntd.0005984.s004.tif]

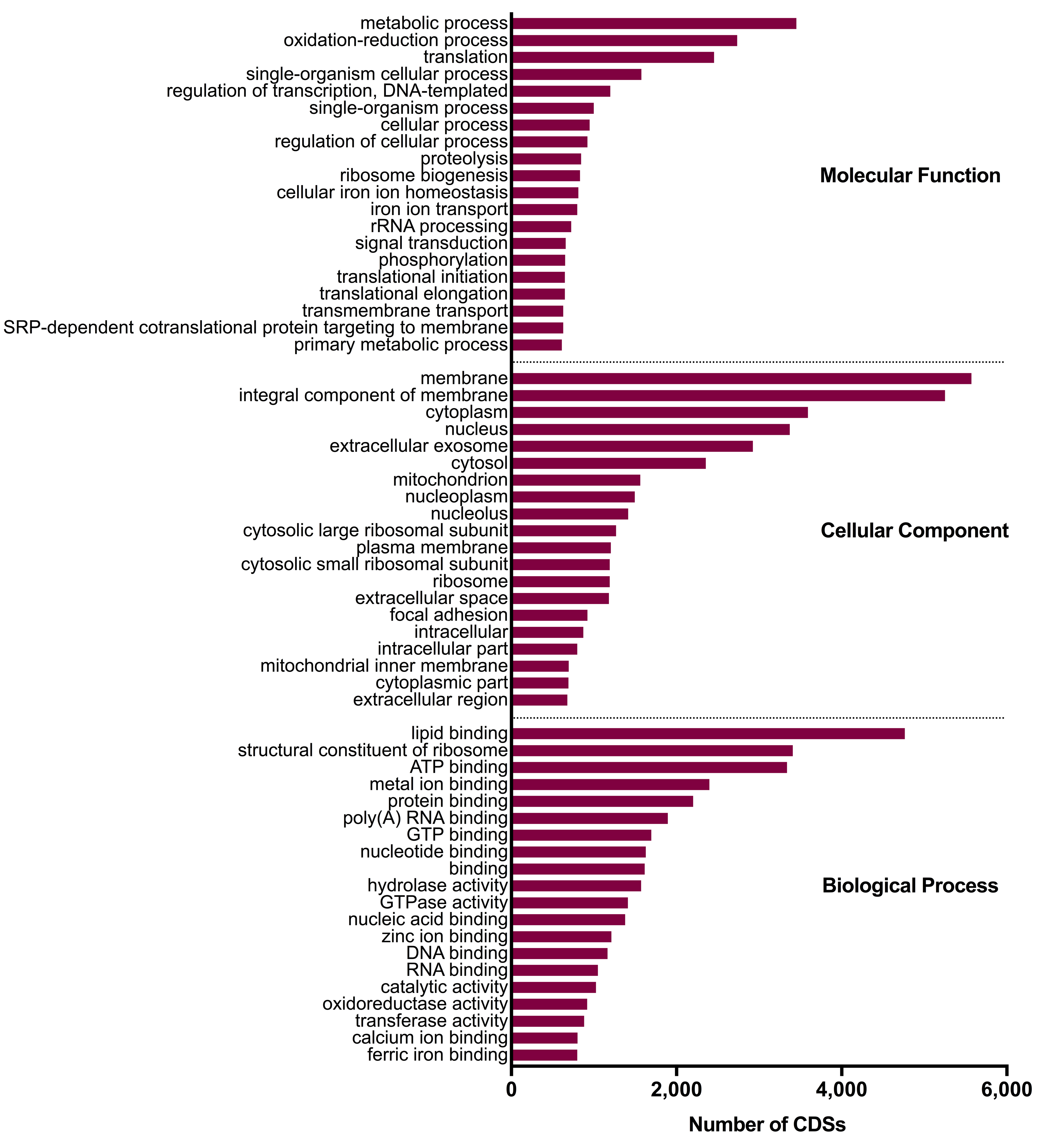

Supplement: S2 Fig — (TIF) [file pntd.0005984.s005.tif]

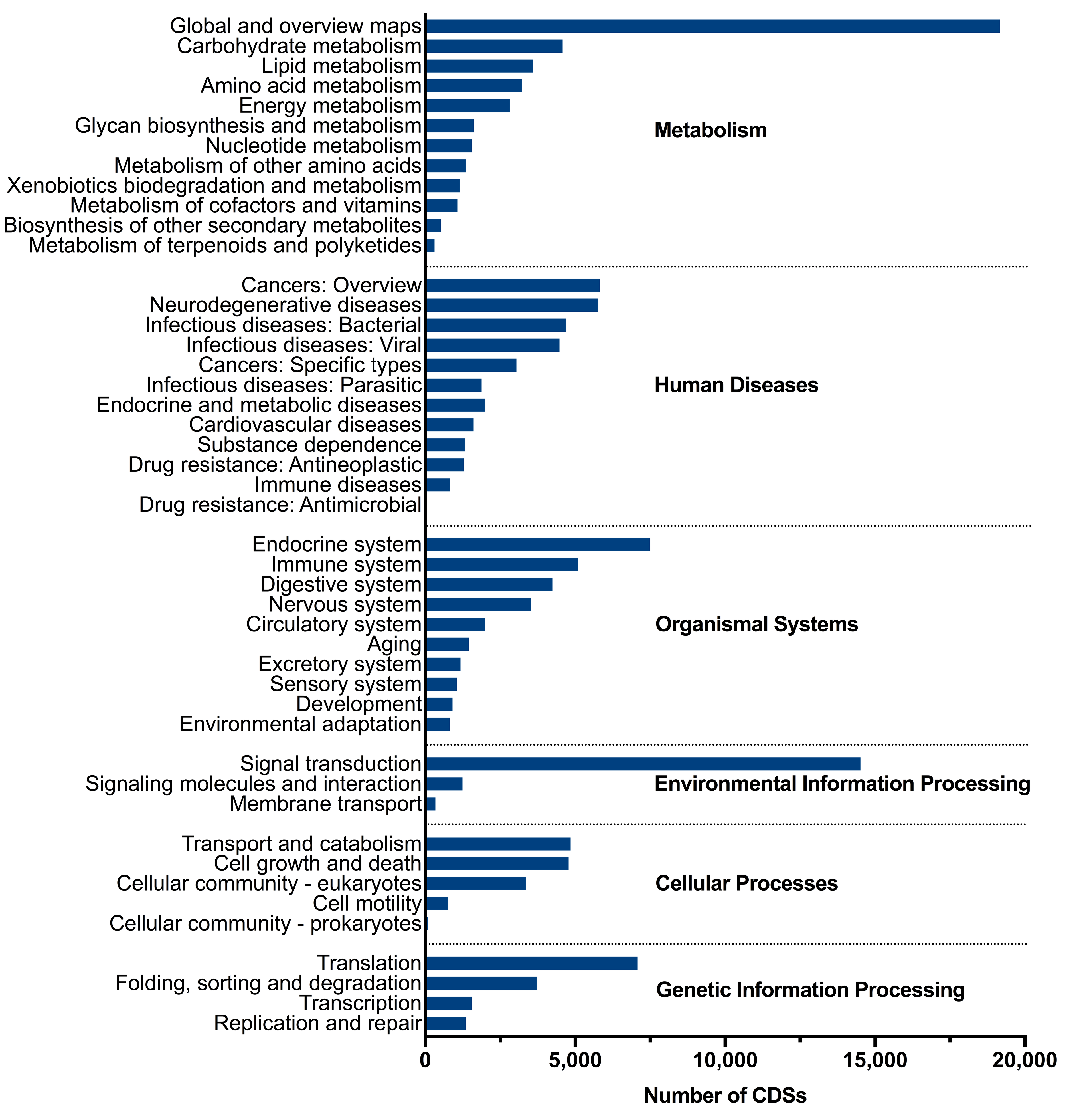

Supplement: S3 Fig — (TIF) [file pntd.0005984.s006.tif]

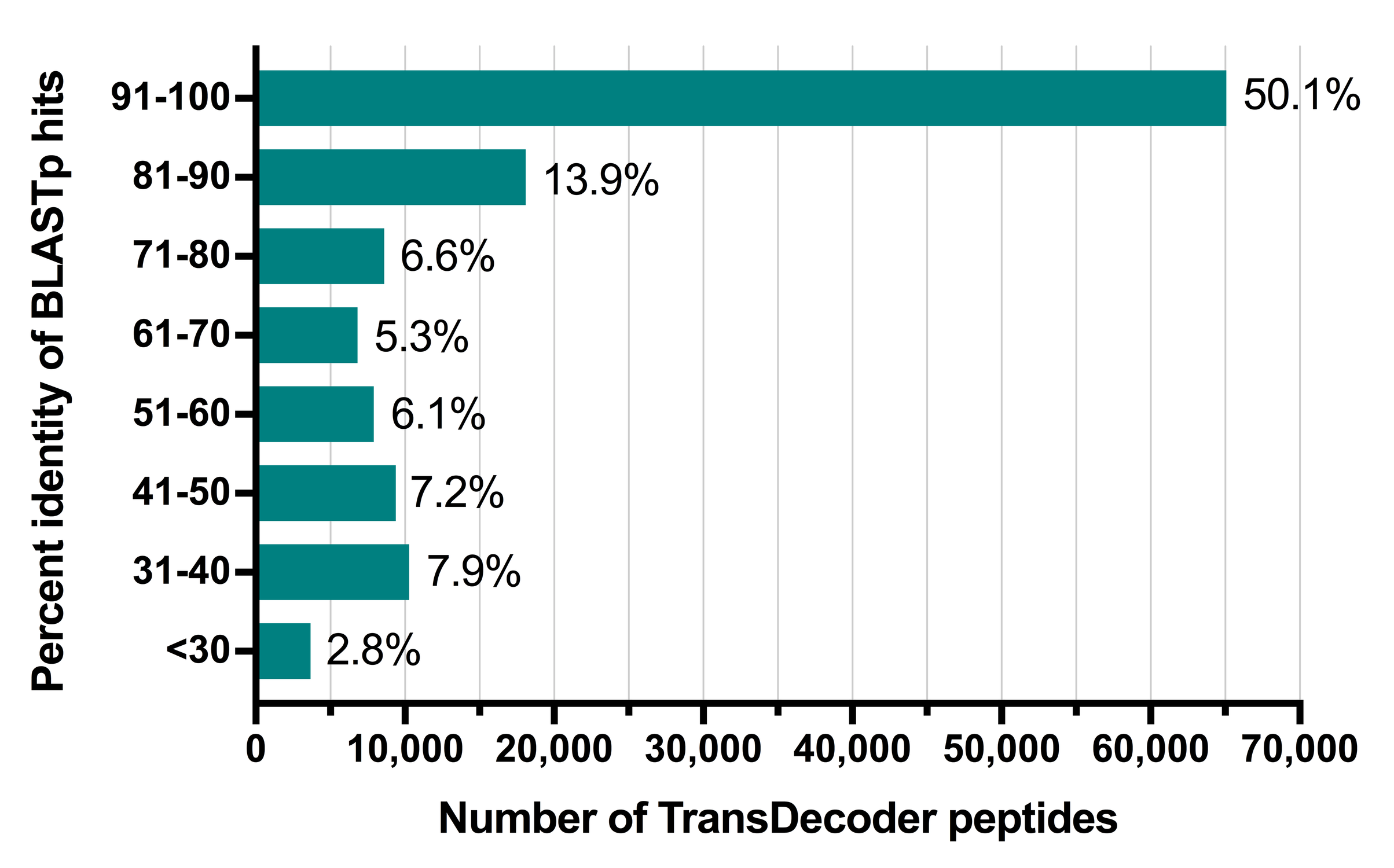

Supplement: S4 Fig — (TIF) [file pntd.0005984.s007.tif]

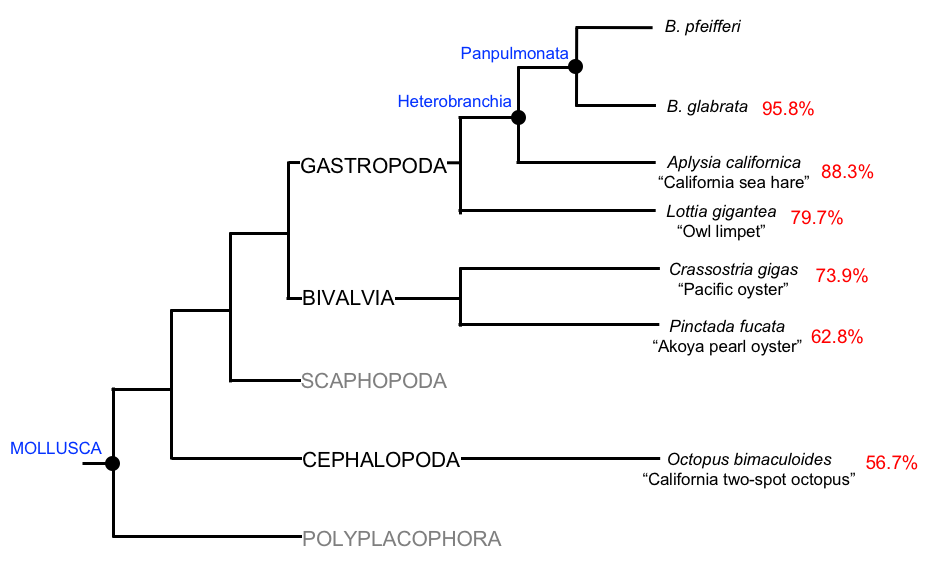

Supplement: S5 Fig — Percentages next to organisms show the percent of proteins present in our B. pfeifferi transcriptome predicted by TransDecoder. (TIF) [file pntd.0005984.s008.tif]
